# Engineering human ventricular heart muscles based on a highly efficient system for purification of human pluripotent stem cell-derived ventricular cardiomyocytes

**DOI:** 10.1186/s13287-017-0651-x

**Published:** 2017-09-29

**Authors:** Bin Li, Hui Yang, Xiaochen Wang, Yongkun Zhan, Wei Sheng, Huanhuan Cai, Haoyang Xin, Qianqian Liang, Ping Zhou, Chao Lu, Ruizhe Qian, Sifeng Chen, Pengyuan Yang, Jianyi Zhang, Weinian Shou, Guoying Huang, Ping Liang, Ning Sun

**Affiliations:** 1 Department of Physiology and Pathophysiology, School of Basic Medical Sciences, State Key Laboratory of Medical Neurobiology, Fudan University, Shanghai, 200032 China; 2 Shanghai Key Laboratory of Clinical Geriatric Medicine, Fudan University, Shanghai, 200032 China; 3 Children’s Hopstital, Fudan University, Shanghai, 201102 China; 4 First Affiliated Hospital, Zhejiang University School of Medicine, Hangzhou, China; 5 Institute of Translational Medicine, Zhejiang University, Hangzhou, 310029 China; 6 Institute of Biomedical Sciences, Fudan University, Shanghai, 200032 China; 7 Department of Biomedical Engineering, University of Alabama, Birmingham, AL 35294 USA; 8 Department of Pediatrics, School of Medicine, Indiana University, Indiana, 46202 USA

**Keywords:** Myosin light chain 2, Myosin light chain 2v, Engineered human heart tissues, Human pluripotent stem cells, Human ventricular cardiomyocytes, Engineered human ventricular heart muscles

## Abstract

**Background:**

Most infarctions occur in the left anterior descending coronary artery and cause myocardium damage of the left ventricle. Although current pluripotent stem cells (PSCs) and directed cardiac differentiation techniques are able to generate fetal-like human cardiomyocytes, isolation of pure ventricular cardiomyocytes has been challenging. For repairing ventricular damage, we aimed to establish a highly efficient purification system to obtain homogeneous ventricular cardiomyocytes and prepare engineered human ventricular heart muscles in a dish.

**Methods:**

The purification system used TALEN-mediated genomic editing techniques to insert the neomycin or EGFP selection marker directly after the myosin light chain 2 (*MYL2*) locus in human pluripotent stem cells. Purified early ventricular cardiomyocytes were estimated by immunofluorescence, fluorescence-activated cell sorting, quantitative PCR, microelectrode array, and patch clamp. In subsequent experiments, the mixture of mature *MYL2*-positive ventricular cardiomyocytes and mesenchymal cells were cocultured with decellularized natural heart matrix. Histological and electrophysiology analyses of the formed tissues were performed 2 weeks later.

**Results:**

Human ventricular cardiomyocytes were efficiently isolated based on the purification system using G418 or flow cytometry selection. When combined with the decellularized natural heart matrix as the scaffold, functional human ventricular heart muscles were prepared in a dish.

**Conclusions:**

These engineered human ventricular muscles can be great tools for regenerative therapy of human ventricular damage as well as drug screening and ventricular-specific disease modeling in the future.

**Electronic supplementary material:**

The online version of this article (doi:10.1186/s13287-017-0651-x) contains supplementary material, which is available to authorized users.

## Background

Myocardial infarction (MI) and subsequent cardiac damage and remodeling remains the leading cause of morbidity and mortality in the world. Owing to the anatomic size of human ventricular heart chambers and the hemodynamic features of the coronary arteries after sclerosis, most MI patients present in the clinics exhibit ventricular myocardium ischemia and dysfunction [1]. Therefore, repairing the damaged ventricular cardiac muscles is the central quest for current cardiac regenerative therapies.

Human pluripotent stem cells (hPSCs), including human embryonic stem cells (hESCs) and human induced pluripotent stem cells (hiPSCs), offered new opportunities for regenerative therapy of human MI [2, 3]. Large-scale production of human cardiomyocytes is now achievable through directed differentiation of hPSCs to the cardiac lineage [4–7], which is able to provide abundant human cardiomyocytes for bioengineering human heart tissues and for cell therapy of myocardial damage. However, cardiac differentiation of hPSCs results in multiple subtypes of cells, including ventricular-like, atrial-like, and sinus nodal-like cardiomyocytes as well as cells of other lineages. Ventricular, atrial, and sinus nodal cardiomyocytes exhibited different physical and electrophysiological properties [8–10]. A mixture of different subtypes of cardiomyocytes may cause multiple sites of ectopic electroactivities and arrhythmia after transplantation into injured myocardium. Indeed, recent studies on transplantation of suspended hESC-derived or hiPSC-derived cardiomyocytes into the ventricles of nonhuman primates eventually led to a different extent of ventricular arrhythmia [11, 12]. Previous studies have made efforts to isolate working-type cardiomyocytes. One study used a molecular beacon targeting working-type CM-associated markers [13]; Karakikes et al. attempted to develop a directed ventricular-like cardiomyocyte differentiation system using small molecules and growth factors [14]. For the purpose of human heart tissue engineering or repairing ventricular muscle damage, using a homogeneous population of ventricular-specific myocytes would be more attractive.

Since currently there are no specific surface markers for ventricular cardiomyocytes, selection of ventricular myocytes usually relies on endogenous genes expressed specifically in ventricular heart muscles or cardiomyocytes. Myosin light chain-2, the regulatory light chain of myosin, is a critical component of the sarcomere of striated muscles. The cardiac ventricular isoform of myosin light chain-2 (MLC-2v, gene name *MYL2*) is restrictively expressed in the ventricular segment of the heart tube at E8.0 in mice and remains exclusively expressed in ventricles until adulthood [15, 16]. *MYL2* is therefore a good genetic locus for driving ventricular-specific gene expression and a specific marker for selection of ventricular cardiomyocytes. Previous studies used either lentivirus-based or adenovirus-based human MYL2 promoter-driving fluorescent protein as a reporter to select hPSC-derived early ventricular cardiomyocytes [9, 17, 18]. They demonstrated that MLC-2v is a robust marker for enriching early ventricular cardiomyocytes after cardiac differentiation of hiPSCs. However, the limitations of a virus-based technique are obvious, which raise safety issues and may not be applicable in future clinical treatment.

In this study, we inserted the neomycin or EGFP selection cassette directly after the *MYL2* gene locus in hPSCs by TALEN-mediated genomic engineering. Using the internal ribosome entry site (IRES) sequence or P2A peptidase signal in between, both markers were coexpressed with the ventricular-specific MLC-2v when cardiomyocytes were differentiated from hPSCs. Highly pure human early ventricular cardiomyocytes can then be enriched by G418 selection or flow cytometry based on the EGFP reporter, which provides a highly efficient system for the purification of hPSC-derived ventricular cardiomyocytes.

Engineered heart tissues (EHTs) represent a promising therapy for myocardium damage in the future. Human ventricular cardiac patches may be more valuable for the treatment of MI since most infractions cause damage in the ventricles. To our knowledge, preparation of human ventricular heart muscles has not been reported to date. With our selected MLC-2v-positive human early ventricular cells, we now are able to generate engineered human ventricular heart muscles in the current study. By combining a fixed ratio of selected hPSC-derived human ventricular cardiomyocytes and mesenchymal cells with pieces of decellularized natural rat heart extracellular matrix (ECM), engineered human ventricular heart muscles of desired shape and size were constructed in a dish. These engineered human ventricular heart muscles have great potential in modeling ventricular heart diseases, in drug screening, as well as for repairing individual-specific ventricular damages in the future.

## Methods

### Construction of TALENs and donor vectors

We designed two targeting donor vectors, tagged by the neomycin resistance cassette (donor 1) or EGFP (donor 2), which are connected by IRES and P2A respectively and therefore driven by the endogenous *MYL2* promoter. To prevent disruption of target gene expression, the tag was inserted in the adjacent intron downstream of the last exon in the *MYL2* gene. We used the internal ribosome entry site (IRES) sequence in between the neomycin coding sequences and the 5' *MYL2* homologous arm in the donor construct. We later found that, for the EGFP donor construct with IRES in between the 5' *MYL2* homologous arm and the EGFP coding sequences, the expression of EGFP was hard to detect after cardiac differentiation of the engineered hESC lines. We therefore used the P2A peptidase signal in between the *MYL2* and EGFP coding sequences. The TAG stop codon of the *MYL2* gene was thus removed in the EGFP donor construct. The 5′-homology arm and the 3′-homology arm of the *MYL2* targeting donor vector were amplified by PCR, introducing overlapping oligonucleotides to facilitate subcloning into the vector.

### Evaluation of the cutting efficiency for TALENs

The 293FT cells were obtained from Shanghai SiDanSai Biotechnology Co. Ltd. Cultured cells were transfected with TALEN plasmids. Genomic DNA was then extracted after 5 days of puromycin selection. The targeted region was amplified for sequencing and T7 Endonuclease I-based Mutation Detection (New England Biolab). Briefly, PCR products were denatured and annealed before T7 Endonuclease I digestion, and finally analyzed by electrophoresis.

### *MYL2* targeting in hPSCs using TALEN-based homologous recombination

The human ESC line H7 used in this study was obtained from WiCell Research Institute under a specific Material Transfer Agreement. The human iPSC line was derived previously from human skin fibroblasts in our laboratory [19] with informed consent approved by the Bioethics Committee of Zhongshan Hospital affiliated to Fudan University. hPSCs cultured on Matrigel (growth factor reduced; BD Biosciences) were disassociated into single cells with Accutase (Invitrogen). Three million cells were electroporated with the TALEN pairs and targeting donor vectors using the Neon Transfection System (Life Technology). Cells were seeded into a Matrigel-coated 10-cm dish containing 8 ml mTeSR1 hPSC Medium (Stem Cell Technology) for recovery at 37 °C with 5% CO_2_. For the selection of transfected cells, 0.25 μg/ml puromycin was used. Single clones were then picked ∼ 2 weeks later to analyze whether the neomycin resistance (donor 1) or EGFP (donor 2) cassette was successfully inserted via homologous recombination using nested PCR followed by gel electrophoresis and confirmed by DNA sequencing. Correctly targeted clones were picked into six-well plates, expanded, and transfected with plasmids expressing Cre-recombinase to excise the loxP-flanked puromycin resistance cassette.

### Teratoma formation

SCID/NOD mice were purchased from Shanghai SLAC Laboratory Animal Co. Ltd. One million undifferentiated hPSCs were suspended in 20 μl Matrigel and injected into the armpit of 8-week-old SCID/NOD mice. All rats were maintained at 24 °C, with free access to food and water. Six weeks after cell delivery, tumors were dissected and fixed with 4% paraformaldehyde in PBS for hematoxylin and eosin (H&E) staining.

### Histology and immunofluorescence staining

Cells were fixed in 4% paraformaldehyde and permeabilized with 0.05% Triton-X100 followed by goat serum blocking. H7 and hiPSC colonies were stained with pluripotency marker antibodies OCT3/4 (Santa Cruz), SOX2 (Abcam), Nanog (Santa Cruz), and SSEA-4 (Abcam), whereas hPSC-derived cardiomyocytes were stained with antibodies for cTNT (Abcam), Sarcomeric α-actinin (Abcam), MYL2 (Proteintech), MYL7 (Synaptic system), or EGFP (Proteintech) for 24 h at 4 °C respectively. Cells were then incubated with Alexa Fluor 594 or 488 at 37 °C for 1 h and subsequently counterstained with DAPI. For rat hearts, heart tissues were paraffin-embedded and sectioned, followed by H&E staining. The remaining tissues were embedded with optimal cutting temperature compound (OCT; Sakura Finetek, Japan) and sectioned into sections 8 mm thick. The slides were then labeled with antibodies for Laminin (Thermo Fisher Scientific), fibronectin (Abcam), and collagen III (Abcam). Images were captured with a fluorescence microscope Leica DMi8 (Leica).

### Cardiac differentiation and culture

Both H7 and hiPSCs were differentiated into the cardiomyocyte lineage following modified protocols described by Lian et al. [6]. Seven to 9 days post differentiation, a beating cluster of cells can be observed, while robust spontaneous contraction occurs by day 10. Cultures were maintained in DMEM with 10% FBS under a 37 °C and 5% CO_2_ air environment.

### G418 selection of MYL2-positive cardiomyocytes

At 22 days post cardiac differentiation, beating cells were digested and seeded into 12-well plates. Then 100 μg/ml G418 (InvivoGen) was used for 7–8 days for the selection of drug-resistant hPSC cardiomyocytes, with medium change every 1–2 days.

### Quantitative PCR analysis

Total RNA was isolated using the Trizol reagent (Life Technologies) and 3 μg total RNA was used to synthesize cDNA using the ReverTra Ace qPCR RT Kit (FSQ-101; TOYOBO) according to the manufacturer's instructions. Quantitative RT-RCR was performed using the SYBR® Green Realtime PCR master mix (TOYOBO) on a CFX96™ Real-Time System instrument (BIO-RAD). Each reaction was run in triplicate to minimize the variation. Gene expression values were normalized to the mean expression of the housekeeping gene GAPDH. Primer sequences are listed in Additional file 1: Table S1.

### Fluorescence-activated cell sorting analysis of hPSC-derived cardiomyocytes

Dissociated cell suspension was filtered with a 40-μm cell strainer (BD Falcon) to remove cell clumps, and the cells were then fixed and permeabilized using BD Cytofix/Cytoperm™ (BD Biosciences) for 30 min at 4 °C. Next, cells were incubated with the primary antibody, including the mouse anti-human TNNT2 antibody (Thermo Scientific) or the rabbit anti-human MYL2 antibody (Proteintech), followed by appropriate FITC or PE-conjugated secondary antibody. Cells were washed twice in BD perm/wash buffer, centrifuged, and resuspended in 200 μl PBS. To define the threshold for positive fluorescence, the isotype control sample was incubated with secondary antibody only. Data were collected using FACSCalibur (BD Biosciences) and analyzed using FlowJo. A total of 10,000 gated events were counted for each marker in three independent experiments.

### EGFP^+^ cell analysis and sorting

For the analysis of EGFP^+^ cardiomyocytes, dissociated and filtered MYL2^EGFP/w^-hPSC-derived cardiomyocytes were resuspended in PBS, and then acquired with the FACSCalibur system (BD Biosciences). GFP-positive cardiomyocytes were sorted using a BD FACSAria II flow cytometer. After the cell sorting procedure, cells were collected and plated as monolayers (~10,000 cells per coverslip) on Matrigel-coated 12-well plates in DMEM with 10% FBS. Medium was changed routinely every 2 days.

### Apoptosis assay

Living cells were washed with ice-cold PBS and labeled with Alexa Fluor 647-conjugated Annexin V and PI, and then incubated for 15 min in the dark as recommended. Cells were detected in 1 h using the FACSCalibur system (BD Biosciences).

### Patch clamp

Cardiac action potentials were recorded in current-clamp mode from single beating cardiomyocytes with the whole-cell patch-clamp technique, using an EPC-10 amplifier (HEKA, Lambrecht, Germany). Data were acquired using PatchMaster software (HEKA) and digitized at 1 kHz. Data analysis was performed using Igor Pro (Wavemetrics, Portland, OR, USA) and Prism (Graphpad, La Jolla, CA, USA). V-like, A-like, and N-like cardiomyocytes were identified by action potential (AP) patterns recorded in normal Tyrode’s solution containing 150 mM NaCl, 5.4 mM KCl, 1 mM MgCl_2_, 15 mM glucose, 1.8 mM CaCl_2_, 1 mM Na-pyruvate, and 15 mM HEPES (pH 7.4 with NaOH). The pipette solution contained 150 mM KCl, 5 mM NaCl, 2 mM CaCl_2_, 10 mM HEPES, 5 mM Mg-ATP, and 5 mM EGTA (pH 7.2 with KOH).

### Neonatal rat ventricular cardiomyocyte isolation and coculture

Neonatal rat ventricular cardiomyocyte (NRVM) cultures were prepared from neonatal 1-day-old Sprague–Dawley rats. The ventricles were finely minced and dissociated with 0.075% collagenase I (Ameresco) and 0.8% Trypsin (Thermo Fisher Scientific) four times, for 10 min each. The resulting cell suspensions were passed through a cell strainer (100-μm mesh pore size; BD Biosciences) to obtain a single cell suspension, and were seeded in 12-well cell culture plates (Corning Life Sciences). For establishing cocultures, on the day of NRVM isolation the hPSC-CMs were counted and mixed with the NRVMs in a ratio of 3:1 before plating. At day 1 of culture, cells were incubated with Brdu (10 μg/ml; Sigma-Aldrich) to inhibit nonmyocyte proliferation.

### Cardiomyocyte maturation culture

Dissociated hPSC-derived cardiomyocytes at 20–21 days post differentiation were plated onto Matrigel-coated six-well plates in mature medium, which consists of RPMI 1640 without glucose (Gibco), 500 μg/ml bovine serum albumin (Yeasen), 213 μg/ml l-ascorbic acid 2-phosphate (Sigma-Aldrich) supplemented with 10 mM d-galactose (Sigma-Aldrich), 4 mM l-lactic acid (Sigma-Aldrich), 1 mM sodium pyruvate (Sigma-Aldrich), 20 μg/ml insulin (Sigma-Aldrich), 1× chemically defined lipid concentrate (Sigma-Aldrich), and 200 ng/ml triiodo-l-thyronine (Sigma-Aldrich). At day 2, the medium was supplemented with 100 ng/ml G418 for another 7 days. From day 9, cells were cultured in mature medium with a medium change every other day.

### Perfusion and decellularization of rat hearts

Hearts were obtained from 200–250 g adult rats and cannulated into the ascending aorta with a blunt 20-gauge needle to perform retrograde coronary perfusion. First, sterile deionized water was perfused for 30 min at 2.0 ml/min, followed by perfusion with 1% sodium dodecyl sulfate (SDS) for 3 h, and 1% triton X-100 with 0.5% EDTA (PH 8.0) for another 30 min. The hearts were then washed with deionized water and phosphate buffered saline (PBS) containing 100 U/ml penicillin (Life Technologies, USA), 100 μg/ml streptomycin (Life Technologies), and 1.25 μg/ml amphotericin B (Sigma-Aldrich) for another 2 h.

### Generation of 3D ventricular heart muscles

The decellularized heart ECM was cut into pieces under sterile conditions. The ECM pieces were then put in wells of 48-well plates as a sheet. The mixture of ventricular cardiomyocytes was then seeded onto the sheet at 10^4^ cells/mm^2^. The ventricular heart muscles were cultured in the mature medium and changed every day.

### Electrophysiological assessment of the ventricular heart muscles

Beating ventricular heart muscles were plated on a 0.1% gelatin-coated microelectrode array (MEA) probe for 2 days with mature medium and then examined by the MEA data acquisition system MEA-2100 (Multi-channel Systems). A 60-channel voltage amplifier system was used for recording ventricular heart muscles. Data analysis was performed with SPIKE2 software (CED.UK).

### Statistical analysis

Data were presented as the SEM of three independent experiments. Student’s *t* test was used to compare two normally distributed data sets. To compare the statistical differences of multiple groups, one-way analysis of variance (ANOVA) was used. *P* < 0.05 was considered statistically significant.

## Results

### Insertion of the neomycin or EGFP selection marker after the MYL2 locus in hESCs by TALEN-mediated homologous recombination

**Fig. 1:**
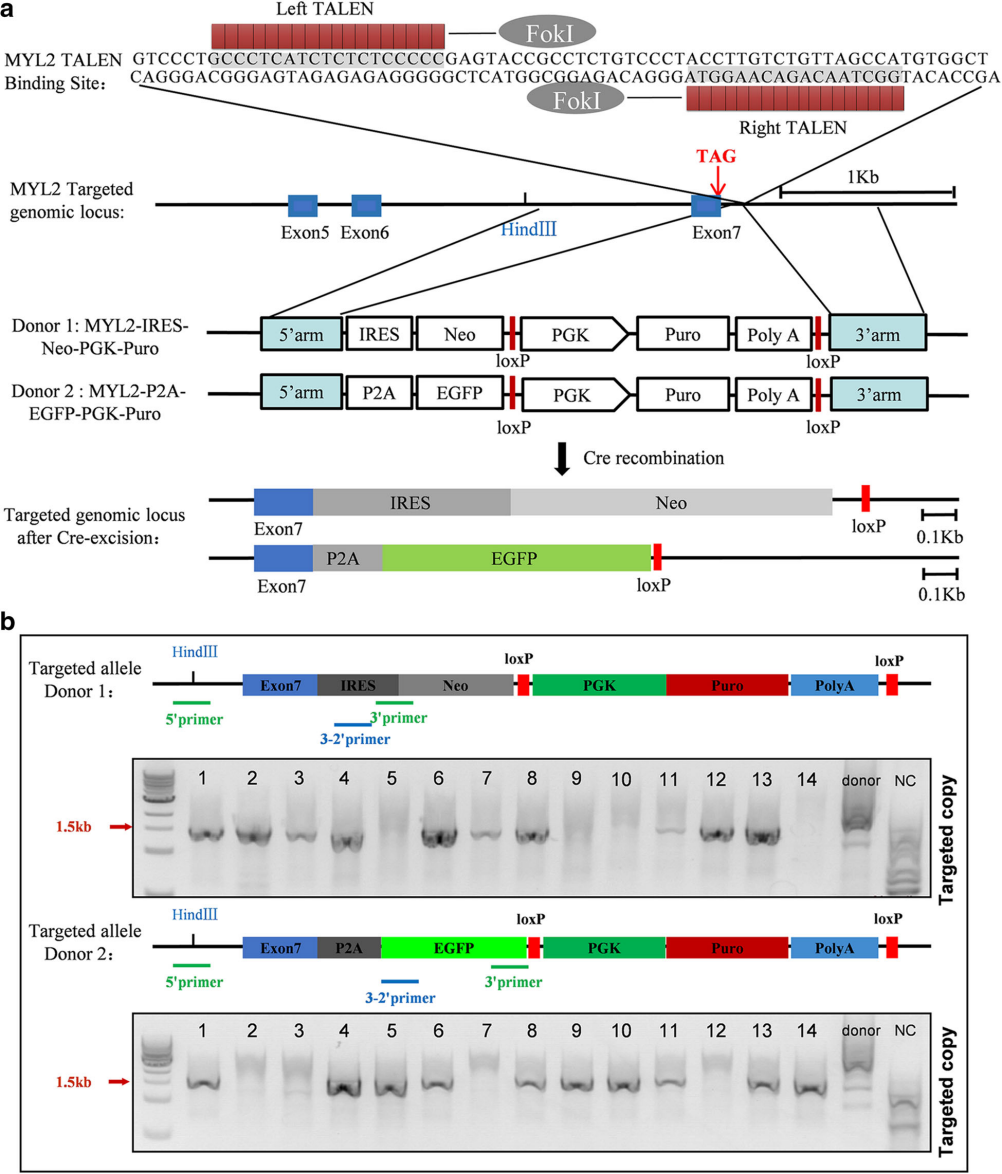
Schematics of the strategy for inserting the neomycin and EGFP selection cassette into the *MYL2* locus. **a** TALENs targeting site for the *MYL2* gene and the homologous recombination events. Red arrow indicates the stop codon of *MYL2* gene. After introduction of a double-strand break near the *MYL2* exon 7 after the stop codon by TALENs, an IRES-Neo (donor 1) or P2A-EGFP (donor 2) along with an excisable PGK-Puromycin drug selection cassette sequence was inserted into the *MYL2* locus downstream of the TAG stop codon (middle panel). The bottom panel shows the targeted genomic locus after Cre-mediated excision of the Puro selection cassette. Blue box, exon of the *MYL2* gene. **b** Nested-PCR strategy to identify successfully targeted clones. Both gels indicated 10 out of 14 clones had at least one copy of the targeted allele (targeting efficiency was 71.5%). *MYL2* myosin light chain 2, TALEN transcription activator-like (TAL) effector nuclease, IRES internal ribosome entry site, P2A self-cleaving peptide sequence, Neo neomycin resistant cassette, EGFP enhanced green fluorescent protein, PGK phosphoglycerol kinase promoter, Puro puromycin resistance gene, polyA polyadenylation sequence, 5’arm 5’-homology arms, 3’arm 3’-homology arms

To insert the neomycin/EGFP selection marker under the C-terminus of the *MYL2* gene located on chromosome 12, we used TALEN-mediated genomic editing techniques in this study. The overall schema of the donor constructs containing the neomycin or EGFP cassette, the specific TALEN cutting sites on the adjacent intron downstream of *MYL2* exon 7, and the homologous recombination events are illustrated in Fig. 1a. The design of the two donor constructs was to allow the neomycin or EGFP to be expressed along with, but separately from, MLC-2v expression after insertion into the 3' terminal of the *MYL2* gene. The purpose of this design was to avoid formation of fusion proteins which may severely affect the normal function of MLC-2v, the myofilaments, and the cardiomyocytes.

We designed and engineered six TALEN pairs (three left (L) TALENs × two right (R) TALENs) targeting downstream of the TAG stop codon on exon 7 of the* MYL2* gene. The L2 + R2 TALEN pair showed a ~20% cutting efficiency by T7EI assays and was confirmed by DNA sequencing (Additional file 2: Figure S1), and thus was used in the subsequent experiments. We next transfected H7 hESCs with the TALEN pair (L2 + R2) and the respective donor construct by electroporation. Positive single clones with successful homologous recombination were then selected with puromycin. Nested PCR using primers flanking the genomic sequence (5' primer) and the donor sequence (3' primer) showed that, in both the neomycin and EGFP groups, 10 out of 14 picked single clones exhibited successful homologous recombination (Fig. 1b). We next transfected these positive clones with Cre recombinase and removed the PGK-puromycin selection cassette. Final DNA sequencing of the genomic sequence of the *MYL2* allele confirmed the correct insertion of the neomycin or EGFP cassette (Additional file 3: Figure S2). To see whether our L2 + R2 TALEN pair caused off-target editing that may cause unwanted mutations at another locus in the genome of these positive clones, we amplified the top 10 potential off-target sites (obtained by TALENoffer software [20]) by genomic PCR and performed DNA sequencing. No off-target editing events were found (Additional file 4: Table S2). We termed these heterozygous hESCs with correct neomycin or EGFP insertion after the *MYL2* gene locus as MYL2^Neo/w^ or MYL2^EGFP/w^ hESCs respectively.

### MYL2^Neo/w^ and MYL2^EGFP/w^ hESCs maintained pluripotency and cardiac differentiation capacities

**Fig. 2:**
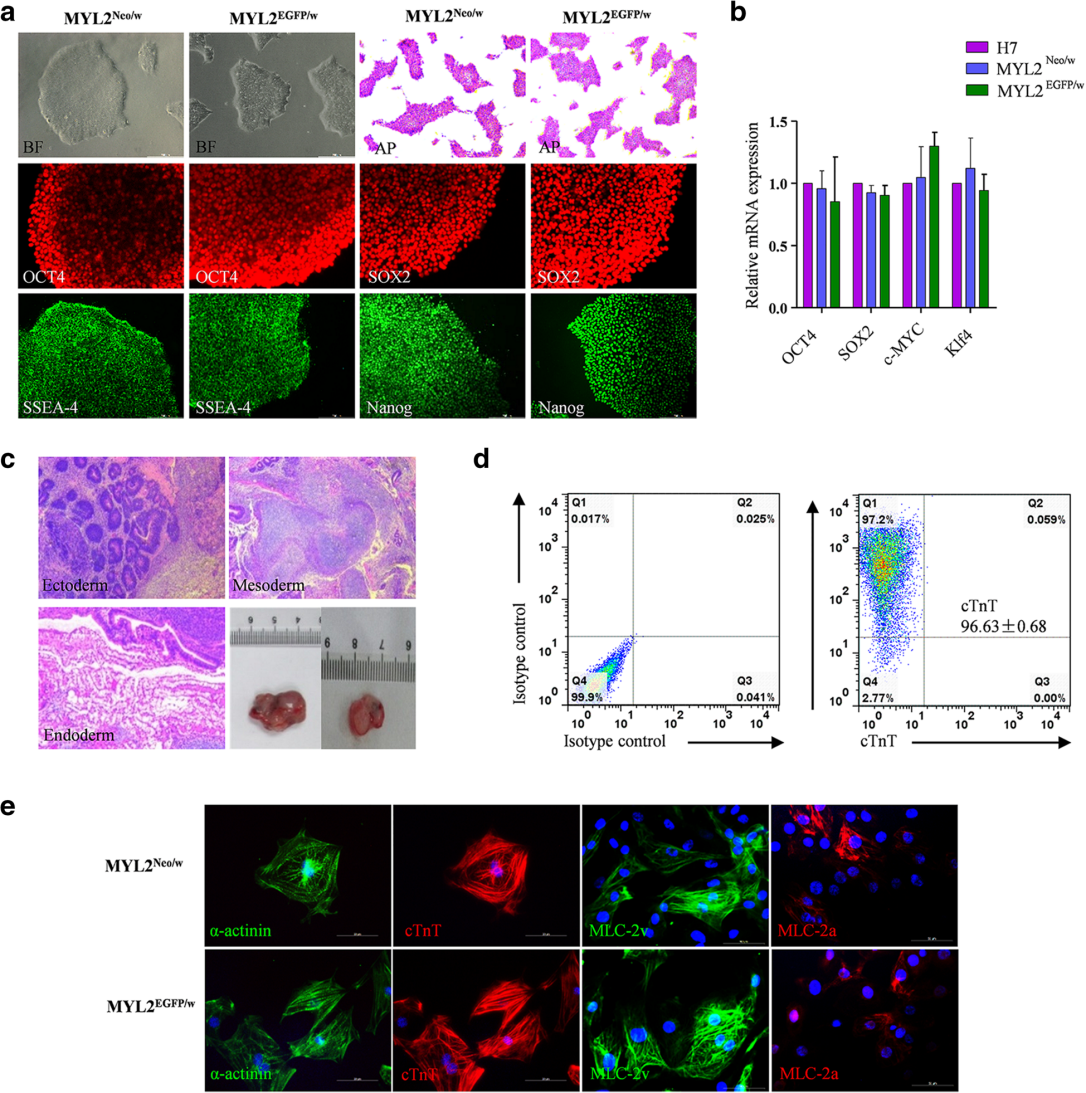
MYL2^Neo/w^ and MYL2^EGFP/w^ hESCs maintained pluripotency and the ability to differentiate into highly pure cardiomyocytes. **a** MYL2^Neo/w^ and MYL2^EGFP/w^ hESCs maintained pluripotent stem cell morphology (bright field (BF)), positive alkaline phosphatase (AP) staining, and expression of the pluripotency markers *OCT4*, *SOX2*, *Nanog*, and *SSEA-4*. Scale bars, 200 μm. **b** Quantitative PCR examining endogenous expression of the pluripotency factors relative to *GAPDH* in MYL2^Neo/w^ and MYL2^EGFP/w^ hESCs. Data obtained from three independent experiments. Wildtype hESCs (H7) used as controls. **c** Undifferentiated MYL2^Neo/w^ and MYL2^EGFP/w^ hESCs formed teratomas, which exhibited tissues of all three developmental germ layers: ectoderm (e.g., sebaceous gland cells), mesoderm (e.g., cartilage cells), and endoderm (e.g., gland cells). Scale bars, 200 μm. **d** Purity of MYL2^Neo/w^ and MYL2^EGFP/w^ hESC-derived cardiomyocytes detected by flow cytometry analysis of cardiac specific marker cTnT. **e** Immunostaining showing MYL2^Neo/w^ and MYL2^EGFP/w^ hESC-derived cardiomyocytes expressed cardiomyocyte specific proteins sarcomeric α-actinin, cTnT, MLC-2v, and MLC-2a. Scale bars, 50 μm. EGFP enhanced green fluorescent protein, MLC-2v cardiac ventricular isoform of myosin light chain-2, MYL2 myosin light chain 2

Under normal culture conditions, MYL2^Neo/w^ and MYL2^EGFP/w^ hESCs maintained typical clonal morphologies and expressed strong alkaline phosphatase activities as normal hESCs did. Immunofluorescence staining showed that these cells expressed pluripotency markers *OCT4*, *SOX2*, *Nanog*, and *SSEA-4* (Fig. 2a). Quantitative PCR also showed that the expression level of the pluripotency genes *OCT4*, *SOX2*, *Klf4*, and *c-MYC* in MYL2^Neo/w^ and MYL2^EGFP/w^ hESCs were similar to those in the wildtype hESCs (H7) (Fig. 2b). Upon injection into immunodeficient mice, MYL2^Neo/w^ and MYL2^EGFP/w^ hESCs formed teratomas containing the three embryonic germ layers (Fig. 2c). These results indicated that insertion of the neomycin or EGFP cassette after the *MYL2* locus in hESCs did not affect their pluripotent potential.

We next differentiated MYL2^Neo/w^ and MYL2^EGFP/w^ hESCs into the cardiac lineage using a modified 2D directed cardiac differentiation protocol [6]. The differentiated cells usually started to beat spontaneously on day 8 or 9 after differentiation (Additional file 5: Video S1). We consistently obtained greater than 90% of the differentiated cells being cTnT-positive cardiomyocytes by fluorescence-activated cell sorting (FACS) analysis in our routine differentiation protocol (Fig. 2d). Further, MLC-2v expression markedly increased along directed cardiac differentiation and become stable after day 25 post differentiation. In addition, neomycin phosphotransferase II and GFP proteins were simultaneously expressed (Additional file 6: Figure S3), indicating again the correct insertion and expression of the selection system. Immunofluorescence staining of the differentiated beating cells showed that they expressed cardiomyocyte-specific markers cTnT, sarcomeric α-actinin, MLC-2v, and MLC-2a (Fig. 2e). These results indicated a successful cardiac differentiation and the correct selection system for the MYL2^Neo/w^ and MYL2^EGFP/w^ hESCs.

### Effective enrichment of MLC-2v-positive human early ventricular cardiomyocytes based on neomycin selection

**Fig. 3:**
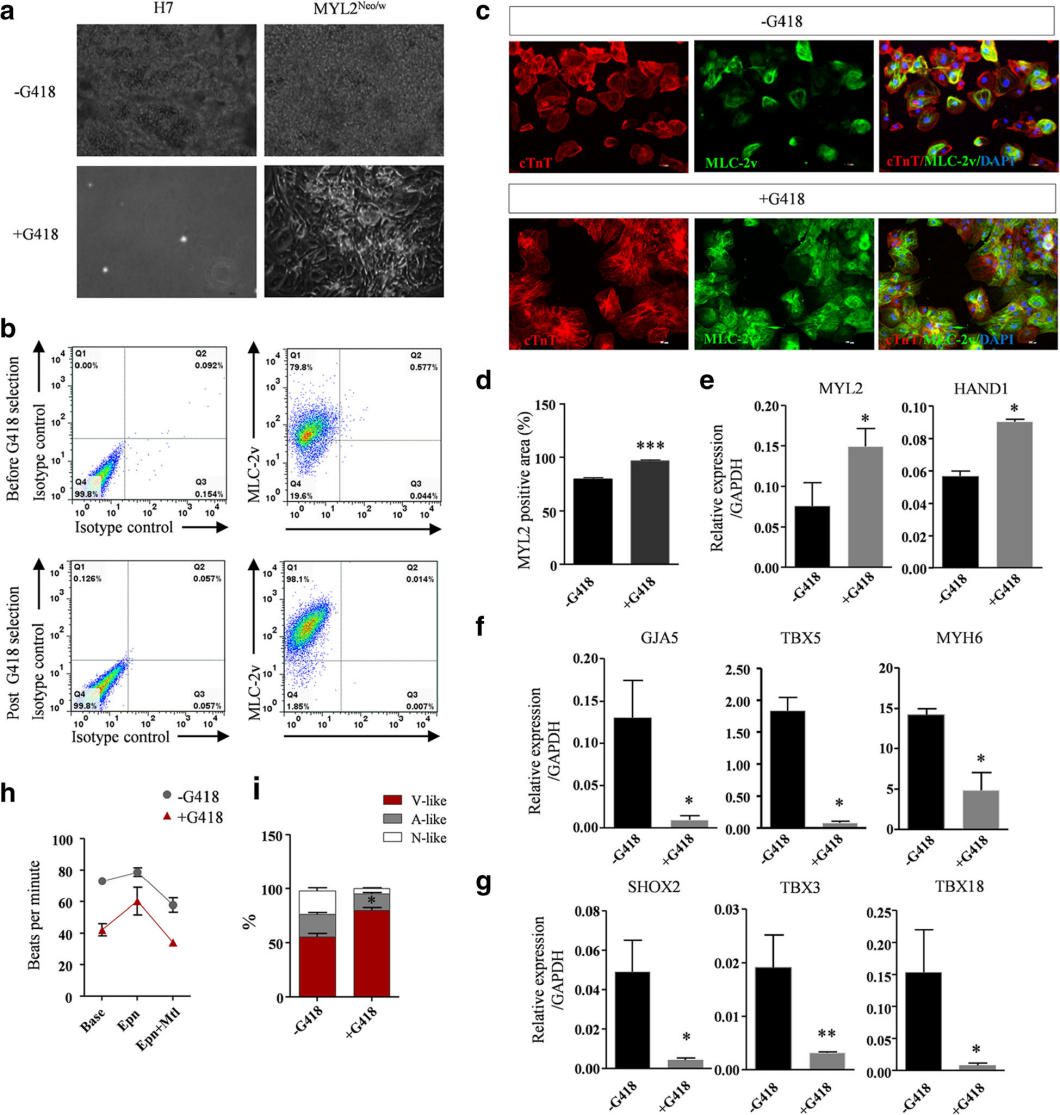
Effective enrichment of MLC-2v-positive human early ventricular cardiomyocytes based on the neomycin selection system. **a** Compared with the wildtype control (H7-derived cardiomyocytes), MYL2^Neo/w^ hESC-derived cardiomyocytes show normal growth following G418 selection for 8–10 days after plating, while control cells died completely. **b** Flow cytometry analysis shows a higher percentage of MLC-2v-positive cells derived from MYL2^Neo/w^ hESCs post G418 selection. **c** Representative immunofluorescence staining images reveal that MLC-2v was expressed in almost all of the G418 selected cTnT-positive MYL2^Neo/w^ hESC-derived cardiomyocytes, while in control cells without G418 selection only partial cells showed MLC-2v expression. Scale bars, 100 μm. **d** Quantification of MLC-2v/cTnT expression ratio in MYL2^Neo/w^ hESC-derived cardiomyocytes before and post G418 selection. *n* = 298, ***P < 0.001 by two-tailed Student’s *t* test. **e**–**g** Quantitative PCR showing expression of ventricular markers *MYL2* and *HAND1* (**e**) was significantly upregulated, while atrial markers *GJA5*, *TBX5*, and *MYH6* (**f**) and nodal markers *SHOX2*, *TBX3*, and *TBX18* (**g**) were significantly reduced in MYL2^Neo/w^ hESC-derived cardiomyocytes post G418 selection. Data shown as the mean ± SEM of three independent experiments. *P < 0.05; **P < 0.01; ns not statistically significant by two-tailed Student’s *t* test. **h** Quantification of beating rates for MYL2^Neo/w^ hESC-derived cardiomyocytes in response to cardiac pharmaceutical reagents before and post G418 selection. **i** Percentages of ventricular-like, atrial-like, and nodal-like cells produced from MYL2^Neo/w^ hESC-derived cardiomyocytes 30 days after cardiac differentiation before and post G418 selection as determined by single cell patch clamp. MYL2 myosin light chain 2, V-like ventricular-like cells, A-like atrial-like cells, N-like nodal-like cells

Compared to cells differentiated from wildtype hESCs (H7) that all died after G418 selection, cardiomyocytes differentiated from MYL2^Neo/w^ hESCs survived and maintained normal cellular growth as well as beating activities under the stress of G418 selection (Fig. 3a and Additional file 7: Video S2). FACS analysis of the differentiated MYL2^Neo/w^ hESCs based on anti-human MLC-2v antibodies indicated that the percentage of MLC-2v-positive cardiomyocytes increased from ~ 80% before G418 selection to > 98% after G418 selection (Fig. 3b). Immunofluorescence staining indicated the percentage of MLC-2v-positive cells over cTnT-positive cardiomyocytes. G418 selection significantly enriched MLC-2v/cTnT double-positive cardiomyocytes derived from MYL2^Neo/w^ hESCs from ~ 78% to ~ 98% (Fig. 3c, d). Quantitative PCR showed ventricular markers *MYL2* and *HAND1* were significantly upregulated (Fig. 3e), while atrial markers *GJA5*, *TBX5*, and *MYH6* (Fig. 3f) and sinus nodal markers *SHOX2*, *TBX3*, and *TBX18* (Fig. 3g) were significantly reduced after G418 selection. Of note, expression of *CX43*, which encodes the predominant cardiac gap-junction protein, was not significantly different with the control (Additional file 8: Figure S4b), indicating that G418 selection did not affect expression of normal junctional proteins of these cardiomyocytes.

Multielectrode array analysis showed that G418-selected MLC-2v/cTnT double-positive cardiomyocytes exhibited normal electrophysiology, longer field potential duration, and lower beating rate, suggesting they were closer to the ventricular form of cardiomyocytes (Fig. 3 h and Additional file 8: Figure S4c, d). We next examined the electrophysiological properties of these MLC-2v-positive cardiomyocytes by whole cell patch clamping. Compared to nonselected MYL2^Neo/w^ hESC-derived cardiomyocytes that contained ~ 55% cells exhibiting ventricular-like action potentials, G418 selection markedly increased the percentage of cells with ventricular-like action potentials and reduced the percentage of cells with atrial-like and nodal-like action potentials (Fig. 3i and Additional file 9: Table S3). Overall, these results indicated that the neomycin selection cassette inserted following the *MYL2* gene is effective in enrichment of MLC-2v-positive early ventricular cardiomyocytes.

### Effective enrichment of MYL2-positive human early ventricular cardiomyocytes based on EGFP selection

**Fig. 4:**
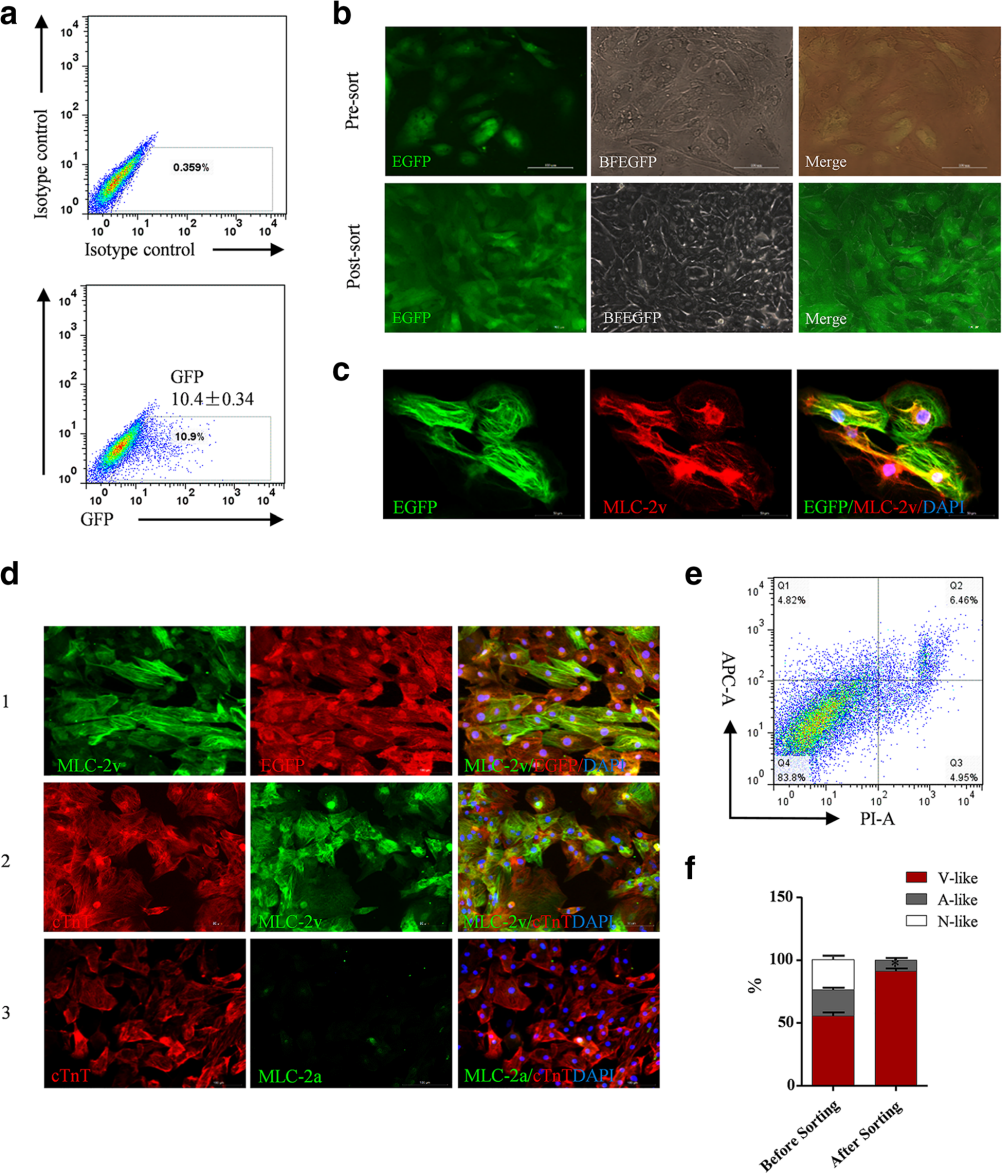
Effective enrichment of MLC-2v-positive human early ventricular cardiomyocytes based on the EGFP selection system. **a** FACS sorting showing positive cardiomyocytes derived from MYL2^EGFP/w^ hESCs 25 days after cardiac differentiation. **b** Representative green fluorescence (EGFP), bright field (BFEGFP), and merged images of MYL2^EGFP/w^ hESC-derived cardiomyocytes before and after FACS sorting. Scale bars, 100 μm. **c** Immunofluorescence microscopy showing coexpression of EGFP and MLC-2v in FACS sorted MYL2^EGFP/w^ hESC-derived cardiomyocytes. Scale bars, 50 μm. **d** Double immunofluorescence staining showing coexpression of EGFP and MLC-2v in FACS sorted MYL2^EGFP/w^ hESC-derived cardiomyocytes (1). MLC-2v was expressed in all of the cTnT-positive MYL2^EGFP/w^ hESC-derived cardiomyocytes post FACS (2). MLC-2a only showed background expression level in the cTnT-positive MYL2^EGFP/w^ hESC-derived cardiomyocytes post FACS (3). Scale bars, 100 μm. **e** Vitality assessment of the FACS selected MYL2^EGFP/w^-CMs by live cell staining for markers of apoptosis. Representative graph as detected by flow cytometry analysis. **f** Percentages of ventricular-like, atrial-like, and nodal-like cells produced from MYL2^EGFP/w^-CMs before and after FACS sorting as determined by single cell patch clamp. EGFP enhanced green fluorescent protein, MLC-2v cardiac ventricular isoform of myosin light chain-2, V-like ventricular-like cells, A-like atrial-like cells, N-like nodal-like cells

We next tested whether genomic EGFP insertion after the *MYL2* gene can be used to enrich MLC-2v-positive early ventricular cardiomyocytes by flow cytometry. FACS showed that there were ~ 10.4% EGFP-positive cells at day 25 post cardiac differentiation of the MYL2^EGFP/w^ hESCs (Fig. 4a). We then sorted out these EGFP-positive cells and maintained them in culture. These cells exhibited relative weak EGFP expressions possibly because of the low copy number due to only one allele insertion of the EGFP cassette in the genome (Fig. 4b). The EGFP-positive cells all showed strong MLC-2v and cTnT expression but very dim MLC-2a expression at the same time (Fig. 4c, d). After FACS selection, MYL2^EGFP/w^-CMs survived well and maintained spontaneous contraction (Additional file 10: Video S3). Assessment of viability showed ~ 83.8% of these cells remained active (Fig. 4e). Whole cell patch clamping showed that almost all of the EGFP-positive cells (~91.3%) exhibited ventricular-like action potentials (Fig. 4f and Additional file 11: Table S4). These results showed that, although the expression is weak, EGFP inserted after the *MYL2* locus is effective to enrich MLC-2v-positive early ventricular cardiomyocytes from cardiac differentiation of hESCs.

### Enrichment of human MLC-2v-positive early ventricular cardiomyocytes from hiPSCs

In the future, hiPSCs could be a powerful tool and widely used for personalized regenerative medicine. To see whether our MYL2^Neo/w^ and MYL2^EGFP/w^ systems also work in hiPSCs as in hESCs, we next transfected hiPSCs generated in our laboratory [19] using the TALEN pair L2/R2 and the respective donor construct by electroporation to insert the neomycin/EGFP selection marker under the C-terminus of the* MYL2* gene. These heterozygous hiPSCs with neomycin or EGFP insertion after the endogenous *MYL2* gene locus were termed MYL2^Neo/w^-hiPSCs and MYL2^EGFP/w^-hiPSCs respectively. As shown in Additional file 12: Figure S5 and Additional file 13: Figure S6, our MYL2^Neo/w^ and MYL2^EGFP/w^ systems also work in hiPSCs.

### Coculture with neonatal rat ventricular myocytes

**Fig. 5:**
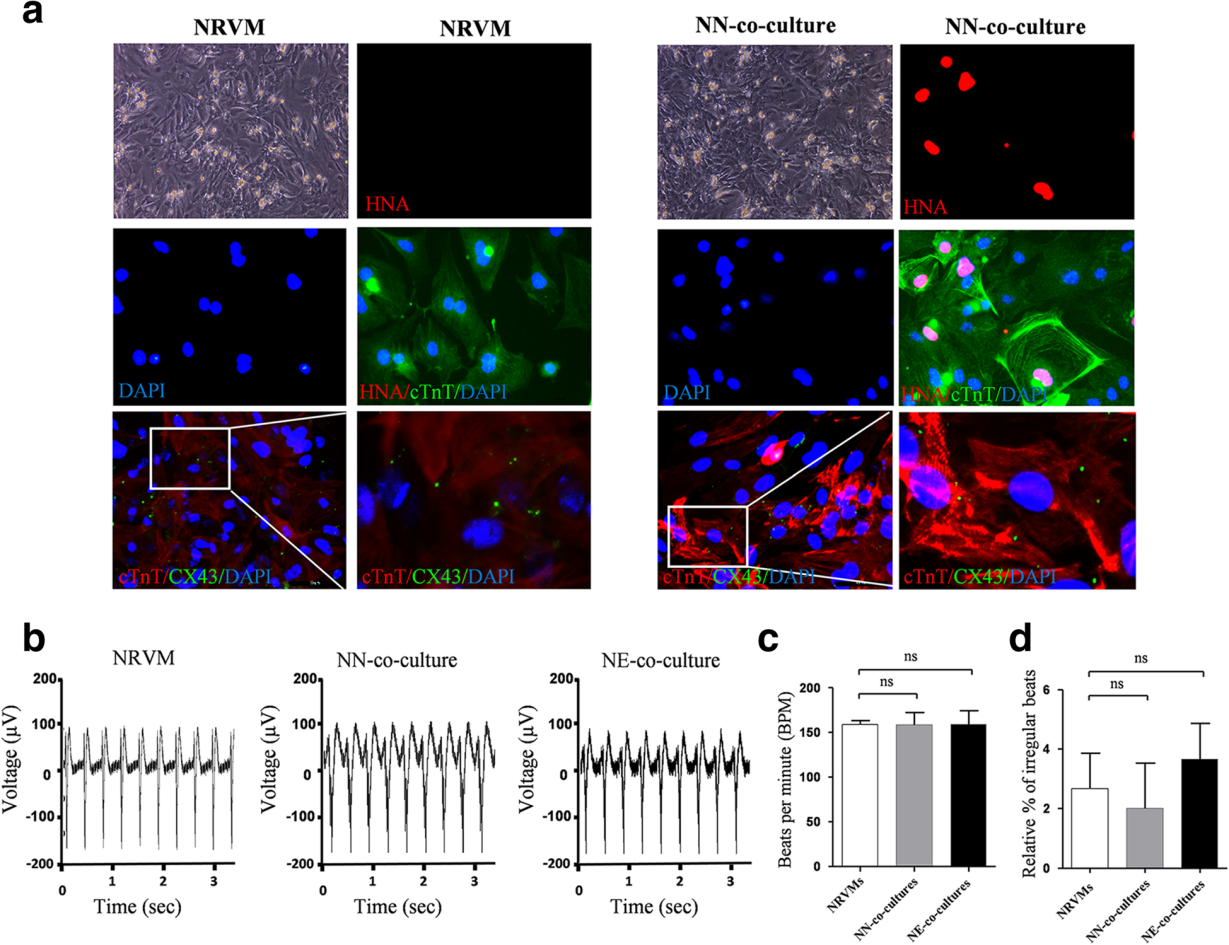
Coculture experiment showing that hPSC-derived ventricular cardiomyocytes electrically coupled with surrounding NRVMs. **a** Representative bright field phase contrast microscopic images (BF) and immunofluorescence staining of NRVM cultures (left panels) and NN-co-cultures, containing NRVMs and G418 selected MYL2^Neo/w^-CMs (right panels) in a 3:1 ratio. Cells were stained for cardiac troponin T (cTnT), human nuclear antigen (HNA), gap-junction protein connexin 43 (CX43), and DAPI. G418 selected MYL2^Neo/w^-CM nuclei were distinguished from rat nuclei by their positive staining for HNA. Scale bars, 50 μm. **b** Typical rhythmic spontaneous field potential recordings in NRVM cultures, NN-co-cultures, and NE-co-cultures (NRVMs and EGFP-positive MYL2^EGFP/w^-CMs). Quantification of (**c**) the beating frequency and (**d**) the percentage of irregular beats. NRVM neonatal rat ventricular myocyte, NN-co-culture MYL2^Neo/w^ cardiomyocyte, NE-co-culture MYL2^EGFP/w^ cardiomyocyte, ns not significant

To investigate the regenerative therapy potential of these hPSC-derived ventricular cardiomyocytes, we cocultured neonatal rat ventricular myocytes (NRVMs) with the selected MYL2^Neo/w^-CMs (NN-co-cultures) or MYL2^EGFP/w^-CMs (NE-co-cultures) in a ratio of 3:1 (Additional file 14: Video S4 and Additional file 15: Video S5). Bright field micrograph showed that NRVMs, as well as NN-co-cultures, formed confluent monolayers. Immunofluorescence staining analyses confirmed a mixture of NRVMs (DAPI^+^ cells) and G418 selected MYL2^Neo/w^-CMs (HNA^+^/DAPI^+^ cells) in NN-co-cultures (Fig. 5a). Importantly, the same as NRVMs, these NN-co-cultures expressed CX43 (Fig. 5a), which play an important role in the formation of gap junctions between ventricular cardiomyocytes, indicating that the cocultures formed cardiac junctions. Next, we performed multielectrode arrays (MEAs) to investigate the synchronous activities of the NN-co-cultures and NE-co-cultures. Both showed regular and synchronous electrical activities similar to NRVM electrophysiological characteristics (Fig. 5b and 5c), Addition of hPSC-derived ventricular cardiomyocytes to NRVMs did not increase their arrhythmogenicity (Fig. 5d), suggesting a well-coupled syncytium of the hPSC-derived ventricular cardiomyocytes and animal heart cells.

### Further maturation of the MLC-2v-positive ventricular-like cardiomyocytes

**Fig. 6:**
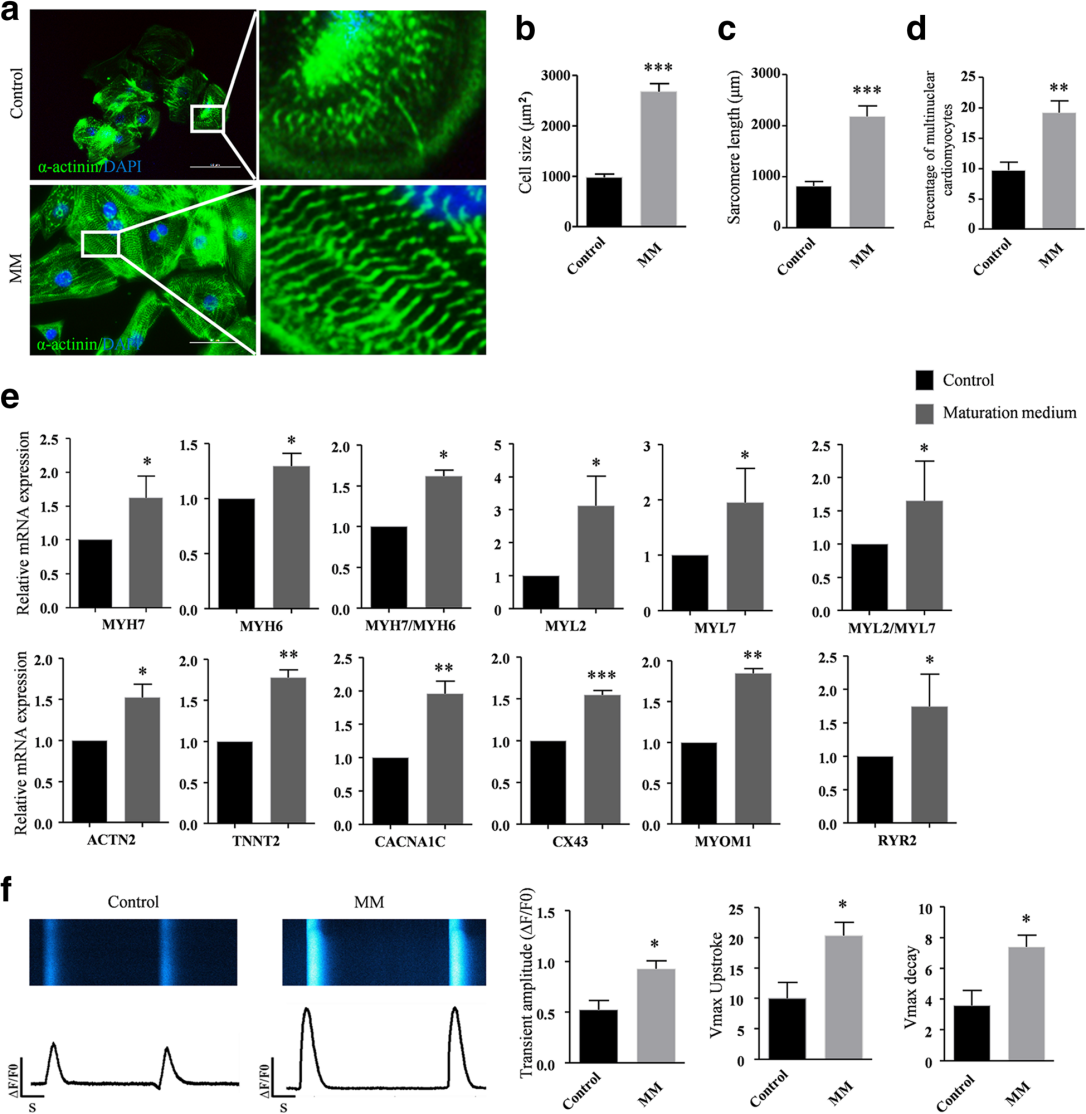
Maturation medium leads to further maturation of the MLC-2v-positive ventricular-like cardiomyocytes. **a** Basal medium (control) and maturation medium (MM)-treated MYL2^Neo/w^-hiPSC-derived cardiomyocytes stained with α-actinin (green) and DAPI (blue). Scale bars, 50 μm. Right panels show enlarged views of the boxed areas in the left merged images, which show detailed α-actinin (green) staining patterns. Compared to MYL2^Neo/w^-hiPSC-derived cardiomyocytes cultured in basal medium (control), MM-treated MYL2^Neo/w^-hiPSC-derived cardiomyocytes show significant changes in cell size (**b**), sarcomere length (**c**), and number of multinuclear cells (**d**). *n* > 100 per condition. **e** Quantitative PCR showing higher transcriptional expression of the functional and mature genes in MM-treated MYL2^Neo/w^-CMs compared with control. Gene expressions shown normalized to *GAPDH*. Data shown as the mean ± SEM of three independent experiments. *P < 0.05; **P < 0.01; ***P <0.001 by two-tailed Student’s *t* test. **f** MYL2^Neo/w^-CMs show larger calcium transient amplitudes, faster upstroke, and faster decay velocities after culture with MM. *n* > 45 per condition

For future regenerative therapy of adult human heart damage such as MI, maturation of the hPSC-derived cardiac cells is a necessary step. In addition, the human ventricular cardiomyocytes selected using our strategy exhibited strong MLC-2v expression and residual MLC-2a expression (Additional file 8: Figure S4a). This pattern was actually consistent with the MLC-2a expression during embryogenesis. Not like MLC-2v is restrictively expressed in the ventricular segment, MLC-2a is uniformly expressed throughout the whole heart tube at E8.0 in mice [21]. With further maturation of the ventricles, MLC-2a expression is downregulated and disappears by E13.5 in ventricular chambers and is only expressed in the atria. Thus, further maturation of the selected MLC-2v-positive cardiomyocytes should better direct them into the ventricular lineage. We thus next sought to further induce maturation of the enriched MLC-2v-positive early ventricular cardiomyocytes [22]. Culturing the MLC-2v-positive cardiomyocytes with the maturation medium greatly enhanced the organization of the myofilaments (Fig. 6a), the cell size (Fig. 6b), and the sarcomere length of these cells (Fig. 6c). The percentage of cells with multiple nuclei, an indicator of cardiomyocyte maturation, also greatly increased (Fig. 6d). Expression levels of functional and mature genes *MYL2*, *MYL7*, *MYH6*, *MYH7*, *TNNT2*, *CX43*, *ACTN2*, *CACNA1C*, *MYOM1*, and *RYR2* were all up-regulated in the further matured *MYL2*-positive cardiomyocytes (Fig. 6e). The ratios of *MYL2*/*MYL7* and *MYH7*/*MYH6,* which indicate maturation of heart tissues, were also significantly increased (Fig. 6e). Calcium transients of the maturation medium-cultured MLC-2v-positive cardiomyocytes were much larger than those of the nonmatured cells. The transient amplitude, maximum upstroke velocity, and maximum decay velocity were all significantly increased (Fig. 6f). All these results demonstrate further maturation of the selected MLC-2v-positive cardiomyocytes.

### Preparation of engineered human ventricular heart muscles using selected MLC-2v-positive ventricular cardiomyocytes

**Fig. 7:**
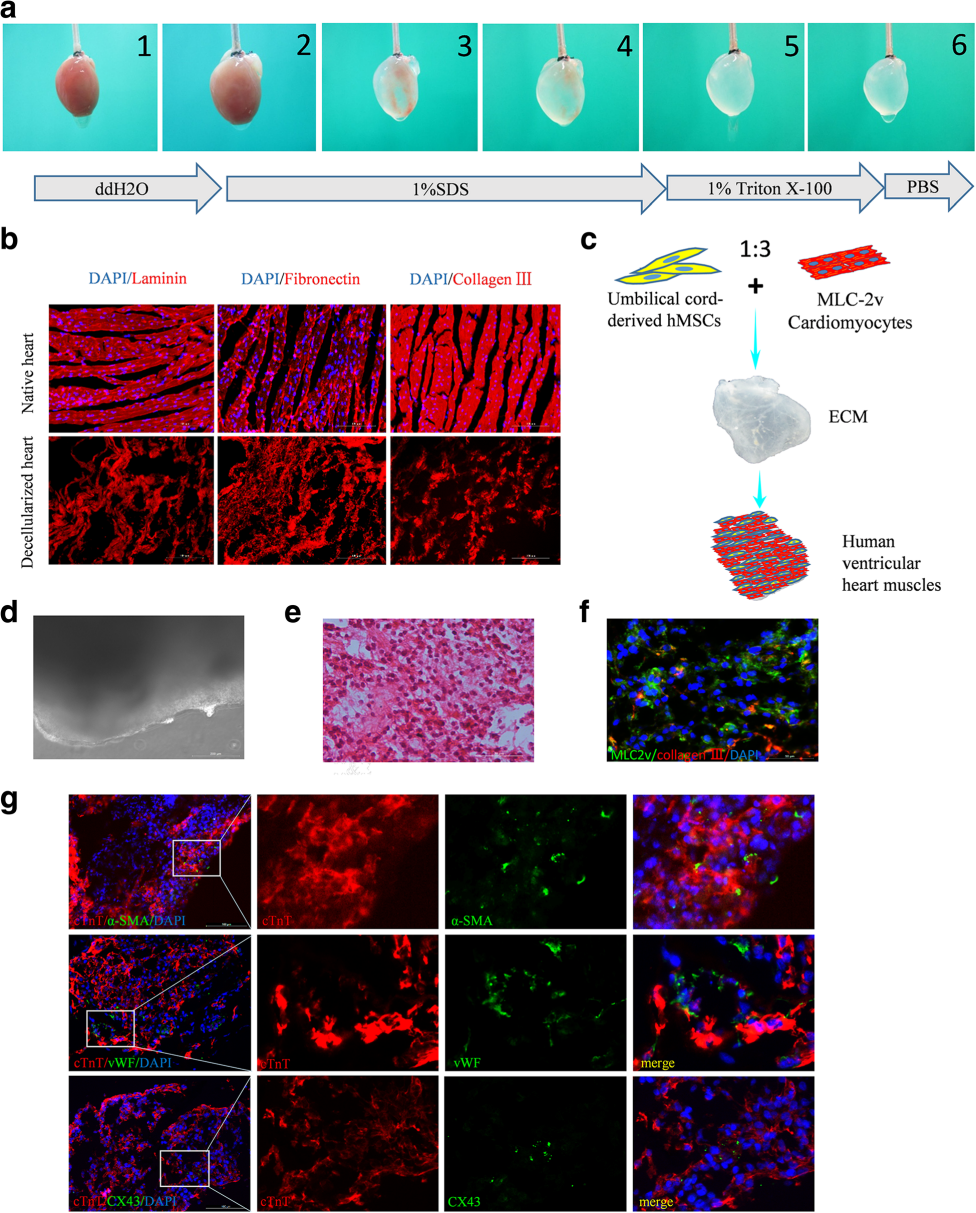
Generation and histological analysis of the human ventricular heart muscles. **a** Process of decellularization of rat hearts and preparation of natural heart ECM. **b** Major ECM compositions (laminin, fibronectin, and collagen III) of decellularized hearts and native rat hearts detected by immunofluorescence staining. **c** Schematic diagram of the preparation of engineered human heart muscles using MLC-2v-positive ventricular cardiomyocytes. **d** Representative image of the engineered human ventricular heart muscles in a light microscope. Scale bar, 200 μm. H&E staining (**e**) and coimmunostaining by anti-MLC-2v and anti-collagen III antibodies (**f**) of sections of constructed ventricular heart muscles. hPSC-derived ventricular cardiomyocytes are well distributed and attached on the native ECM. Scale bar, 100 μm. **g** Double immunofluorescence staining for cTnT/α-smooth muscle actin (α-SMA), cTnT/von Willebrand factor (vWF), and cTnT/CX43 in the engineered human ventricular heart muscles. Scale bars, 100 μm. ECM extracellular matrix, MLC-2v cardiac ventricular isoform of myosin light chain-2, hMSCs human mesenchymal stem cells

For the scaffold of the engineered tissues, we used decellularized natural heart ECM [19, 23, 24] by perfusion of native rat cadaveric heart with detergents (Fig. 7a). Immunofluorescence staining was used to detect ECM compositions. Laminin, fibronectin, and collagen III, which are major proteins of the ECM, remained present within the decellularized heart matrices after removal of cells (Fig. 7b). Nonmyocytes such as fibroblasts are also another indispensable component for bioengineered heart tissues [25]. In this study, we used mesenchymal cells derived from Warton's jelly of the human umbilical cord as the nonmyocyte component [26]. To yield optimal structural and functional properties for EHTs [27], we used the combination of hPSC-derived 75% ventricular cardiomyocytes and 25% umbilical cord-derived nonmyocytes (Fig. 7c). The mixture of cells on pieces of decellularized native rat heart ECM formed a compact tissue-like structure after 2 weeks (Fig. 7d and Additional file 16: Figure S7). H&E and immunofluorescence staining indicated that the mixed cardiac cells attached and grew well on the native heart matrix (Fig. 7e, f). Immunofluorescence staining for the coexpression of cTnT and α-SMA or vWF indicated the newly formed tissues contained cardiomyocytes, smooth muscle cells, and endothelial cells, which are the primary cell types in human adult heart tissues (Fig. 7g). However, expression of CD31 was not detectable (data not shown), suggesting that mature vessels were not fully formed at this moment. Meanwhile, cardiac gap-junction protein connexin 43 (CX43) was present at numerous points of contact between adjacent cells, which promote cell–cell contact and rapid electrical transmission for the heart tissues (Fig. 7g).

**Fig. 8:**
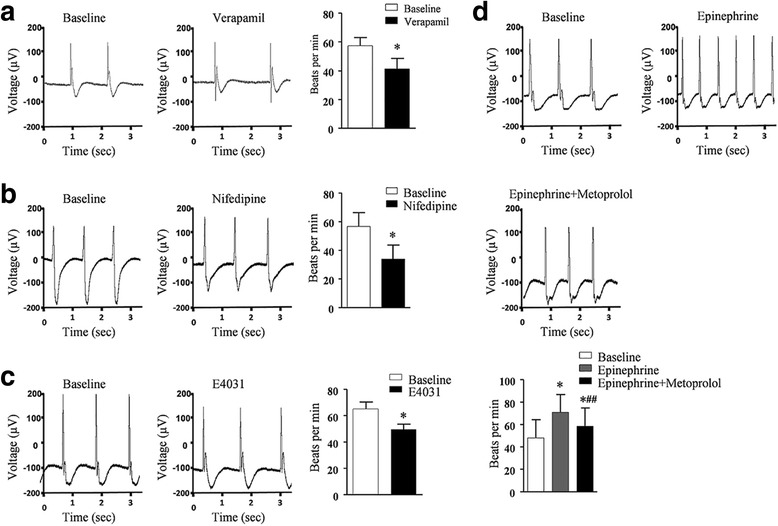
Electrophysiology analysis of human ventricular heart muscles. **a**–**d** Ventricular heart muscles exhibited normal electrophysiology and responded to cardiac pharmaceutical reagents. MEA recording of the field potentials and contraction rate of the engineered human heart muscles before (baseline) and after treatment of verapamil (**a**), nifedipine (**b**), E4031 (**c**), and epinephrine and metoprolol (**d**). Data shown as the mean ± SEM of three independent experiments. *P < 0.05 compared with baseline; *##*P* < 0.05 compared with epinephrine alone by two-tailed Student’s *t* test

These engineered ventricular heart muscles exhibited spontaneous contractile and electrophysiological activities (Additional file 17: Video S6) and responded well to pharmaceutical reagents verapamil (Fig. 8a), nifedipine (Fig. 8b), E4031 (Fig. 8c), and epinepherine/metoprolol (Fig. 8d) in vitro.

## Discussion

Obtaining a pure population of human ventricular cardiomyocytes would greatly strengthen human heart tissue engineering and regenerative therapy for ventricular damage. Using TALEN-mediated genomic engineering, we have successfully inserted the neomycin and EGFP selection markers directly after the ventricular-specific *MYL2* gene within the genome of hPSCs. In this way, neomycin or EGFP was coexpressed with MLC-2v in a separate form when hPSCs were differentiated into cardiomyocytes. Based on these markers, we were able to enrich highly pure human early ventricular cardiomyocytes from hPSC cardiac differentiation and use them for construction of engineered human ventricular heart muscles.

Previous studies used a different strategy than ours to select hiPSC-derived early ventricular myocytes. They produced either lentiviruses or adenoviruses carrying *MYL2* promoter-driving fluorescent proteins, generated stable infected hPSC lines or infected hiPSC-derived cardiomyocytes, and then collected the MLC-2v-positive cardiomyocytes by FACS [17, 18, 28]. However, there are certain limitations of virus-based techniques: the virus infection rate is usually low in hiPSC-derived human cardiomyocytes and this will lead to a waste of many noninfected cardiomyocytes [17]; and using viruses raises safety issues and causes the problem of ectopic genomic insertion and leak expression, which further compromises the purity of selected ventricular cells and their future clinical use. Our method avoided the disadvantages of virus-based strategies and will be more suitable for future clinical applications.

In terms of selection efficiency, our neomycin-based drug selection strategy yielded a very high efficiency and purity (~99%) of MLC-2v-positive hPSC-derived cardiomyocytes. The EGFP-based selection strategy yielded a ~ 10.4% efficiency in sorting pure MLC-2v-positive cardiomyocytes from cardiac differentiation of hPSCs by FACS. This could be due to the relative weak EGFP expressions because of only one allele insertion of the EGFP cassette after the *MYL2* locus. Homozygous insertion of the EGFP cassette may further increase the EGFP expression level as well as the resulting selection efficiency and should be examined in the future. Of note, a recent pioneer study developed strategies for selection of mouse ESC-derived ventricular-like cardiomyocytes based on molecular beacons (MBs) targeting the ventricular specific gene *IRX4* [29]. However, the transfection efficiency of the MBs targeting *IRX4* in human PSC-derived cardiomyocytes was not reported and remains to be investigated. Our neomycin-based selection strategy exhibited ~ 99% efficiency in enrichment of human MLC-2v-positive ventricular cardiomyocytes and avoided steps of FACS in the MB method, which further reduced the chance of contamination by flow cytometry.

By combining decellularized native heart matrix as the scaffold, the selected pure hPSC-derived early ventricular cardiomyocytes, and mesenchymal cells derived from Warton's jelly of human umbilical cord as the nonmyocyte component, we further constructed pieces of engineered human ventricular heart muscles with different sizes and shapes. These engineered human ventricular heart muscle constructs responded well to pharmaceutical agents specific to heart physiologies including epinephrine, nifedipine, E4031, and verapamil. Of note, although we used 75% ventricular myocytes and 25% nonmyocytes [27] to make these constructs, the optimal percentage of myocytes and nonmyocytes should be determined in the future. The treatment effect of these human ventricular heart muscles for ventricular myocardial damages should be further tested. In the future, it is feasible to use our method to construct functional individual-specific ventricular cardiac patches based on hPSCs for ventricular disease modeling, drug screening, as well as personalized regenerative therapy for ventricular damage.

## Conclusions

This study provides a novel and effective system for isolating hPSC-derived ventricular cardiomyocytes by introducing genetic constructs into hPSCs using TALEN-mediated genomic editing. Clinical-scale homogeneous human ventricular-specific cardiomyocytes could be isolated based on G418 selection or flow cytometry. When combined with promoting maturation and using decellularized natural heart ECM as the scaffold, our study for the first time constructed functional engineered human ventricular muscles of desired shape and size in a dish. Overall we believe these engineered human ventricular heart muscles should have great potential in cardiac transplantation studies and cardiovascular research.

## Additional files

Additional file 1: Table S1. Presenting quantitative real-time PCR primers. (DOCX 13 kb)

Additional file 2: Figure S1. Showing TALEN pairs designed for targeting the MYL2 gene locus and the evaluation of TALEN-mediated cutting efficiencies. **a** Schematic diagram of six TALEN pairs designed for the endogenous MYL2 gene. **b** Sanger sequencing of the PCR amplified genomic. **c** TALEN-mediated cutting efficiencies of the targeted locus measured by the T7EI assays. Frequency of gene disruption of the two selected TALEN pairs indicated below each lane. Percentage of indels quantified by ImageJ software. (JPG 175 kb)

Additional file 3: Figure S2. Showing genomic PCR of the targeted locus of MYL2 and DNA sequencing. Sequencing results of the MYL2 targeted locus (from 5′ side) in the positive clones show in-frame correct positioning of the MYL2 neomycin drug selection (**a**) and EGFP reporter (**b**) cassette. No indels were detected in the clones. Furthermore, 3′ end also shows in-frame positioning of the cassette without any indels (JPG 367 kb)

Additional file 4: Table S2. Presenting summary of off-target analysis for the MYL2 targeting locus. Ten potential TALEN off-target sites were estimated by the TALENoffer software. All 10 sites were amplified by PCR and sequenced. (DOCX 16 kb)

Additional file 5: Video S1. showing spontaneous beating of hPSC-derived cardiomyocytes observed 8–9 days after directed cardiac differentiation of hPSCs. (MP4 7630 kb)

Additional file 6: Figure S3. Showing the targeted clones presented changed MLC-2v expression and effective neomycin or EGFP expression. **a** Real-time PCR examining MLC-2v expression change post cardiomyocyte differentiation. Data were mean of three experimental replicates. **b** Western blot analysis of the MYL2^Neo/w^ hESC-derived cardiomyocytes expressing neomycin phosphotransferase II (Neo) (upper panel) and MYL2^EGFP/w^ hESC-derived cardiomyocytes expressing EGFP protein (lower panel). (JPG 82 kb)

Additional file 7: Video S2. Showing cardiomyocytes differentiated from MYL2^Neo/w^ hESCs maintained cellular growth and beating activities under the stress of G418 selection. (MP4 18000 kb)

Additional file 8: Figure S4. Showing the MYL2^Neo/w^ system was effective in selection of MLC-2v-positive cardiomyocytes. **a** Compared with controls, immunostaining for MLC-2a showed a background level after G418 selection. Nuclei were stained with DAPI (blue). Scale bars, 100 μm. **b** Expression of CX43 was comparable before and after G418 selection. **c** Representative traces of MEA showing field potentials (heart beats) recorded in MYL2^Neo/w^ hESC-derived cardiomyocytes before and post G418 selection, followed by application of a β-adrenergic agonist (Epineprine (Epn), 10 μM) and antagonist (Metoprolol (Mtl), 100 μM). **d** Quantification of the beating rate of MYL2^Neo/w^-hPSC-derived ventricular cardiomyocytes (vCMs) and hPSC-derived wildtype cardiomyocytes (CMs) cultured in DMEM with 10% FBS. (JPG 238 kb)

Additional file 9: Table S3. Presenting whole-cell patch clamp recordings of action potentials of ventricular-like, atrial-like, and nodal-like cells produced from day 30 MYL2^Neo/w^-derived cardiomyocytes before and post G418 selection. (DOCX 17 kb)

Additional file 10: Video S3. Showing FACS selected MYL2^EGFP/w^-CMs maintained cellular growth and spontaneous beating. (MP4 2520 kb)

Additional file 11: Table S4. Presenting whole-cell patch clamp recordings of action potentials of ventricular-like, atrial-like, and nodal-like cells produced from day 30 MYL2^EGFP/w^-derived cardiomyocytes before and post FACS sorting. (DOCX 16 kb)

Additional file 12: Figure S5. Showing analyses of pluripotency in representative hiPSC clones. **a**, **b** MYL2^Neo/w^-hiPSCs and MYL2^EGFP/w^-hiPSCs exhibited similar morphologies, alkaline phosphatase activities, and expression levels for the pluripotent genes OCT4, SOX2, Klf4, and c-MYC to those in wildtype hiPSCs. Scale bars, 200 μm. (JPG 266 kb)

Additional file 13: Figure S6. Showing enrichment of human MLC-2v-positive early ventricular cardiomyocytes from hiPSCs. **a**, **b** Adding G418 after cardiac differentiation of MYL2^Neo/w^-hiPSCs successfully selected cardiomyocytes and markedly increased the percentage of MLC-2v-positive cells to ~ 98%. **c** Immunostaining also showed that, after G418 selection, MLC-2v-positive cardiomyocytes were enriched while MLC-2a-positive cardiomyocytes were almost completely removed. **d** FACS sorting based on GFP expression after cardiac differentiation of MYL2^EGFP/w^-hiPSCs successfully enriched pure MLC-2v-positive early ventricular cardiomyocytes. Almost 100% of these EGFP^+^ cells showed strong MLC-2v expression in cTnT-positive cardiomyocytes. (JPG 475 kb)

Additional file 14: Video S4. Showing a typical video of NN-co-cultures under phase-contrast microscopy. (MP4 2200 kb)

Additional file 15: Video S5. Showing a typical video overlapping bright field and EGFP images of NE-co-cultures. (MP4 1580 kb)

Additional file 16: Figure S7. Showing the different sizes and shapes of the engineered ventricular muscles. (JPG 31 kb)

Additional file 17: Video S6. Showing a representative video showing an engineered human ventricular heart muscles cultured on multielectrode arrays. (MP4 3040 kb)

## Abbreviations

CM: Cardiomyocyte
DMEM: Dulbecco Modified Eagle Medium
ECM: Extracellular matrix
EGFP: Enhanced green fluorescent protein
EHT: Engineered heart tissue
FBS: Fetal bovine serum
H&E: Hematoxylin and eosin staining
hESC: Human embryonic stem cell
hiPSC: Human induced pluripotent stem cell
hMSCs: human mesenchymal stem cells
hPSC: Human pluripotent stem cell
IRES: Internal ribosome entry site
MEA: Microelectrode array
MI: Myocardial infarction
MLC-2v: Cardiac ventricular isoform of myosin light chain-2
MYL2: Myosin light chain 2
NRVM: Neonatal rat ventricular myocyte
PSC: Pluripotent stem cell
RT-RCR: Reverse transcription PCR
SEM: Standard error of the mean
TALEN: Transcription activator-like (TAL) effector nuclease
