# Supplementary material for: Engineering human ventricular heart muscles based on a highly efficient system for purification of human pluripotent stem cell-derived ventricular cardiomyocytes
Source: Stem Cell Res Ther. 2017 Sep 29;8:202. doi: 10.1186/s13287-017-0651-x (PMC5622416; doi:10.1186/s13287-017-0651-x)
Supplement: Supplementary file 1 — Presenting quantitative real-time PCR primers. (DOCX 13 kb) [file 13287_2017_651_MOESM1_ESM.docx]

| Primer | Forward | Reverse |
| --- | --- | --- |
| MYH6 | GCTGGTCACCAACAATCCCTA | CGTCAAAGGCACTATCGGTGG |
| MYH7 | ACTGCCGAGACCGAGTATG | GCGATCCTTGAGGTTGTAGAGC |
| TBX3 | GGACCTCTGATGAGTCCTCCA | TCGCTGGGACATAAATCTTTGAG |
| SHOX2 | CAAAGAGGATGCGAAAGGGAT | AGTGGGTCTCGTCAAAAAGCC |
| KCNA5 | CGCGTCCACATCAACATCTC | GGTAGAAGCGTATCTCGTCCG |
| GJA5 | GCTGCCAGAATGTCTGCTAC | GGTACTCGTAAGAGCCAGAGC |
| TBX5 | CTGTGGCTAAAATTCCACGAAGT | GTGATCGTCGGCAGGTACAAT |
| MYL2 | TTGGGCGAGTGAACGTGAAAA | CCGAACGTAATCAGCCTTCAG |
| MYL7 | GCCCAACGTGGTTCTTCCAA | CTCCTCCTCTGGGACACTC |
| CX43 | CAATCTCTCATGTGCGCTTCT | GGCAACCTTGAGTTCTTCCTCT |
| TNNT2 | GGAGGAGTCCAAACCAAAGCC | TCAAAGTCCACTCTCTCTCCATC |
| ACTN2 | CAAACCTGACCGGGGAAAAAT | CTGAATAGCAAAGCGAAGGATGA |
| CACNA1C | TGATTCCAACGCCACCAATTC | GAGGAGTCCATAGGCGATTACT |
| MYOM1 | GAGTCGATATGGGATGCACAC | TCCTTTAACATTCATCGCCGAG |
| RYR2 | CATCGAACACTCCTCTACGGA | GGACACGCTAACTAAGATGAGGT |
