# Supplementary figures and images for: Engineering human ventricular heart muscles based on a highly efficient system for purification of human pluripotent stem cell-derived ventricular cardiomyocytes
Source: Stem Cell Res Ther. 2017 Sep 29;8:202. doi: 10.1186/s13287-017-0651-x (PMC5622416; doi:10.1186/s13287-017-0651-x)

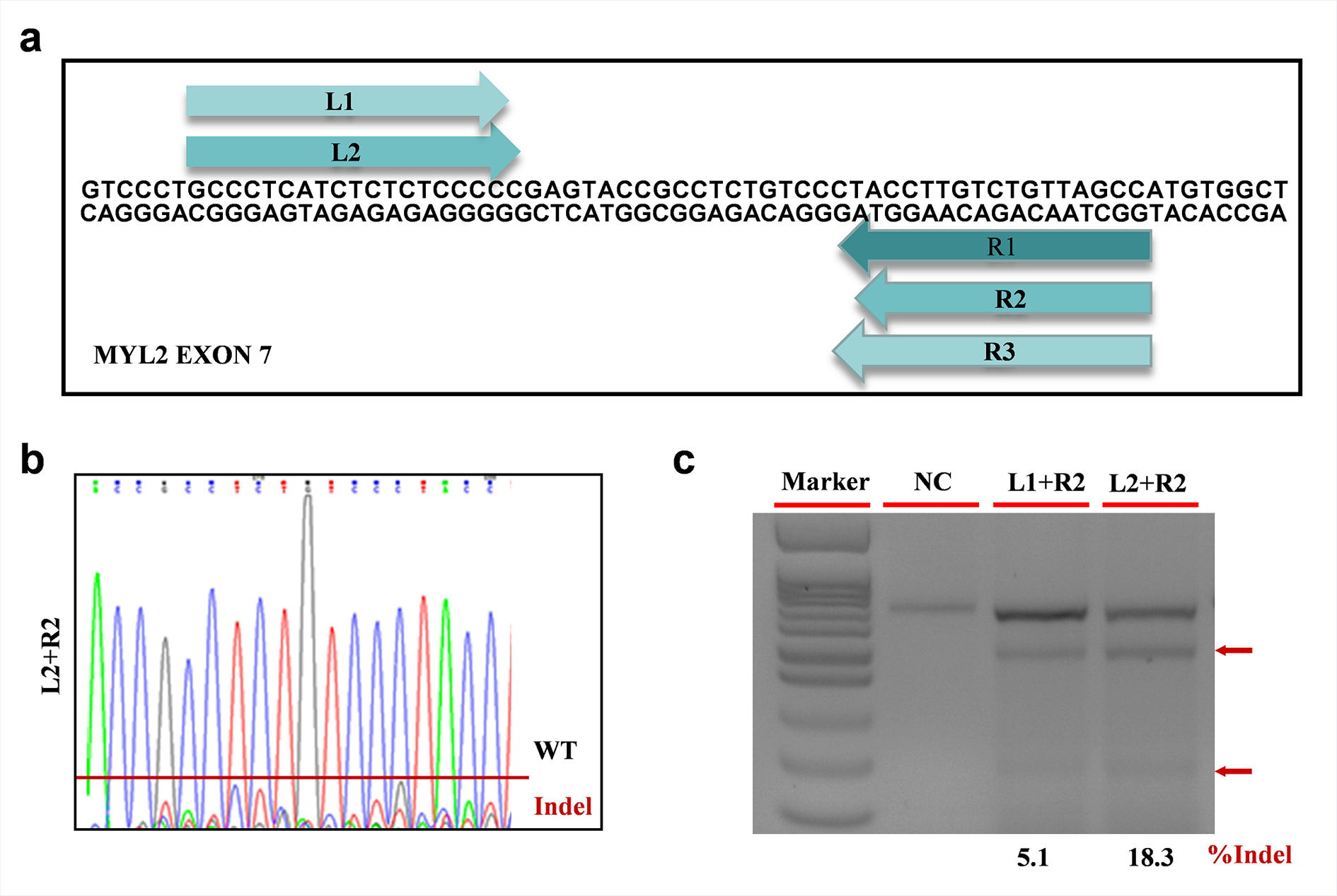

Supplement: Supplementary file 2 — Showing TALEN pairs designed for targeting the MYL2 gene locus and the evaluation of TALEN-mediated cutting efficiencies. a Schematic diagram of six TALEN pairs designed for the endogenous MYL2 gene. b Sanger sequencing of the PCR amplified genomic. c TALEN-mediated cutting efficiencies of the targeted locus measured by the T7EI assays. Frequency of gene disruption of the two selected TALEN pairs indicated below each lane. Percentage of indels quantified by ImageJ software. (JPG 175 kb) [file 13287_2017_651_MOESM2_ESM.jpg]

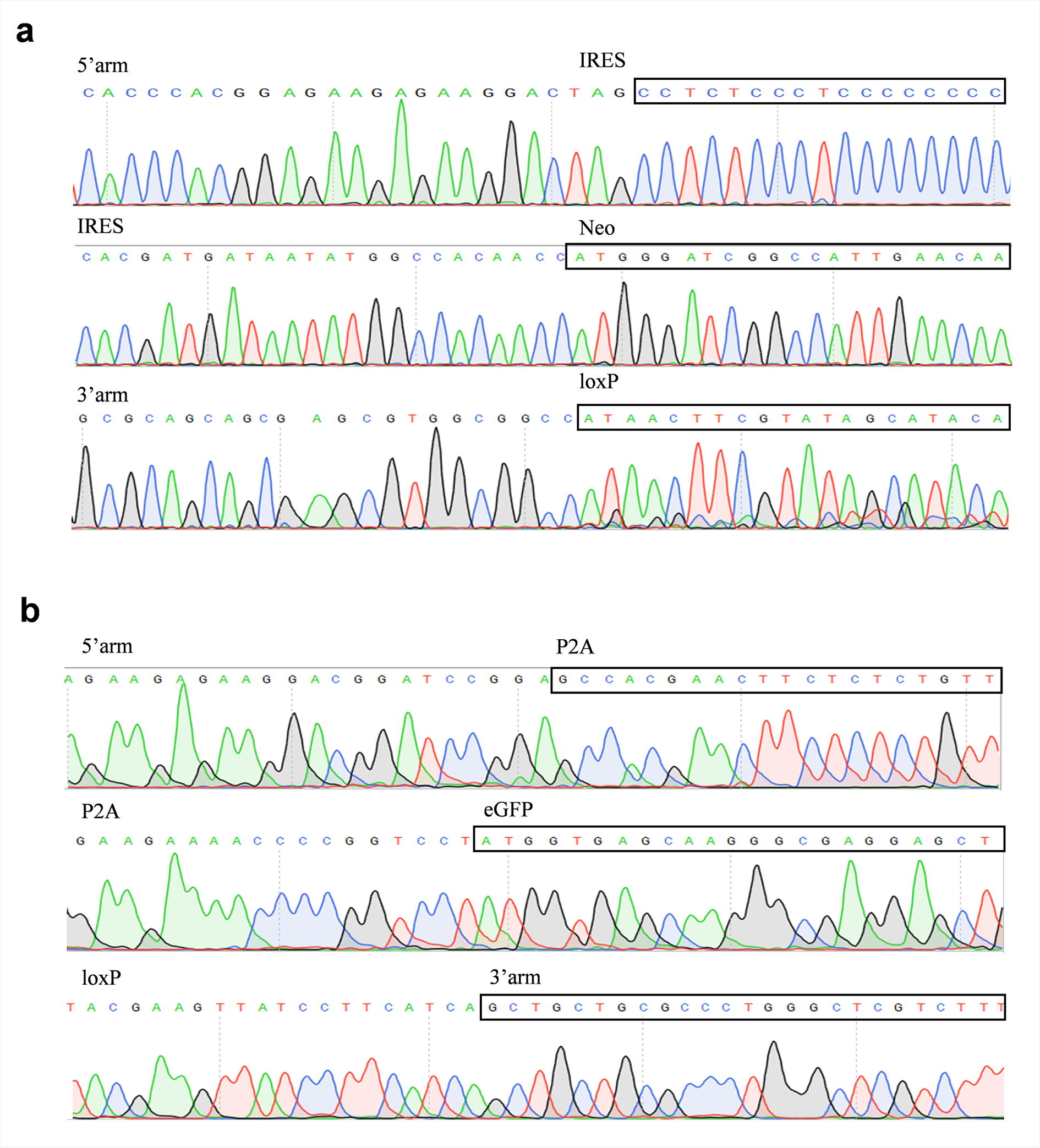

Supplement: Supplementary file 3 — Showing genomic PCR of the targeted locus of MYL2 and DNA sequencing. Sequencing results of the MYL2 targeted locus (from 5′ side) in the positive clones show in-frame correct positioning of the MYL2 neomycin drug selection (a) and EGFP reporter (b) cassette. No indels were detected in the clones. Furthermore, 3′ end also shows in-frame positioning of the cassette without any indels (JPG 367 kb) [file 13287_2017_651_MOESM3_ESM.jpg]

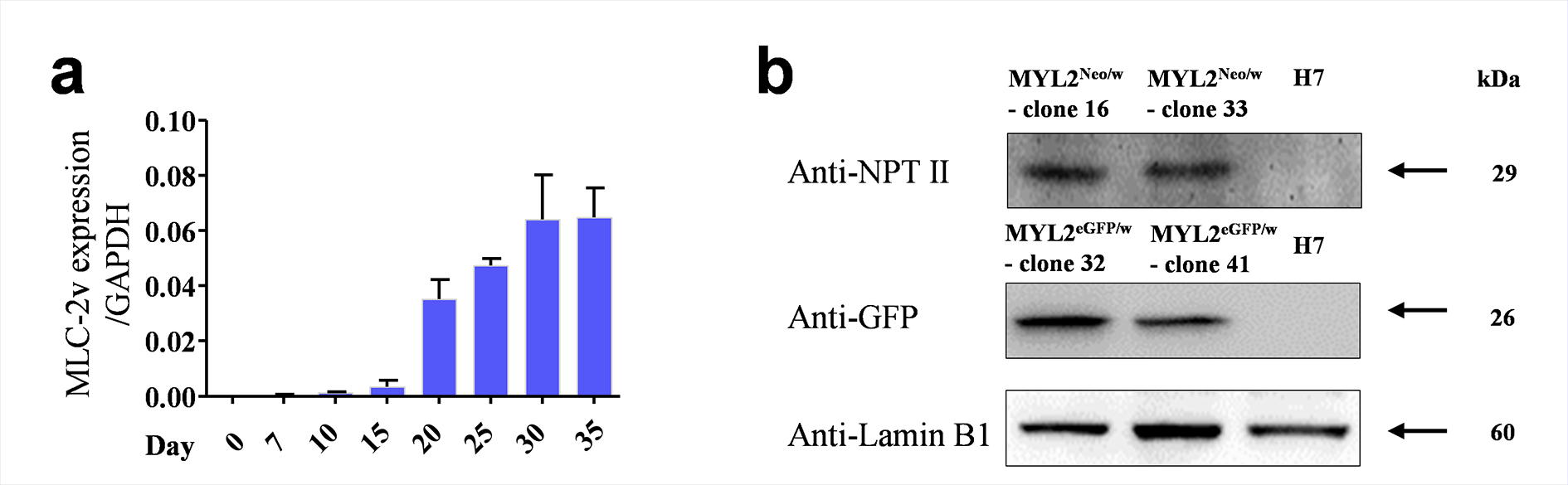

Supplement: Supplementary file 6 — Showing the targeted clones presented changed MLC-2v expression and effective neomycin or EGFP expression. a Real-time PCR examining MLC-2v expression change post cardiomyocyte differentiation. Data were mean of three experimental replicates. b Western blot analysis of the MYL2Neo/w hESC-derived cardiomyocytes expressing neomycin phosphotransferase II (Neo) (upper panel) and MYL2EGFP/w hESC-derived cardiomyocytes expressing EGFP protein (lower panel). (JPG 82 kb) [file 13287_2017_651_MOESM6_ESM.jpg]

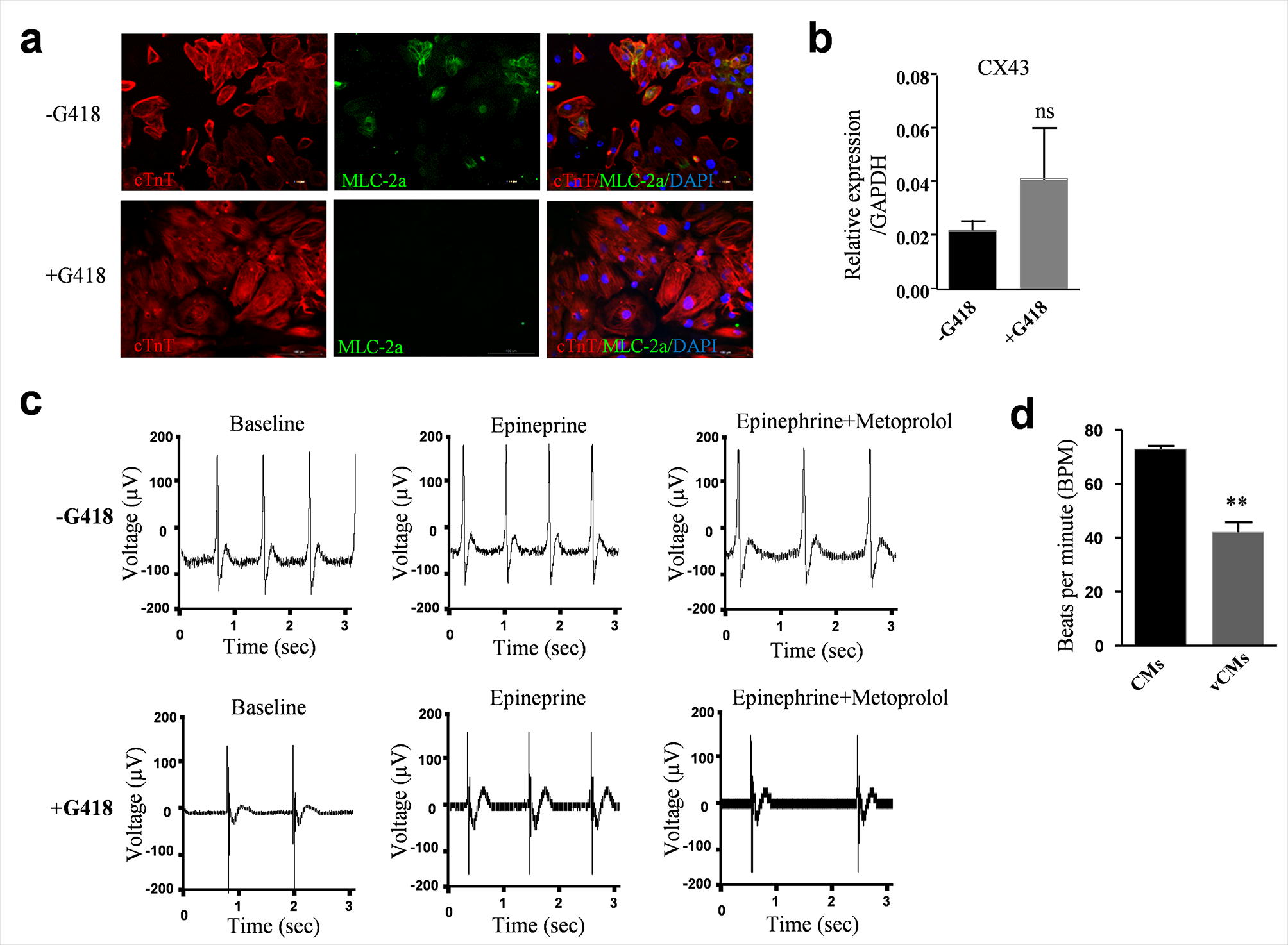

Supplement: Supplementary file 8 — Showing the MYL2Neo/w system was effective in selection of MLC-2v-positive cardiomyocytes. a Compared with controls, immunostaining for MLC-2a showed a background level after G418 selection. Nuclei were stained with DAPI (blue). Scale bars, 100 μm. b Expression of CX43 was comparable before and after G418 selection. c Representative traces of MEA showing field potentials (heart beats) recorded in MYL2Neo/w hESC-derived cardiomyocytes before and post G418 selection, followed by application of a β-adrenergic agonist (Epineprine (Epn), 10 μM) and antagonist (Metoprolol (Mtl), 100 μM). d Quantification of the beating rate of MYL2Neo/w-hPSC-derived ventricular cardiomyocytes (vCMs) and hPSC-derived wildtype cardiomyocytes (CMs) cultured in DMEM with 10% FBS. (JPG 238 kb) [file 13287_2017_651_MOESM8_ESM.jpg]

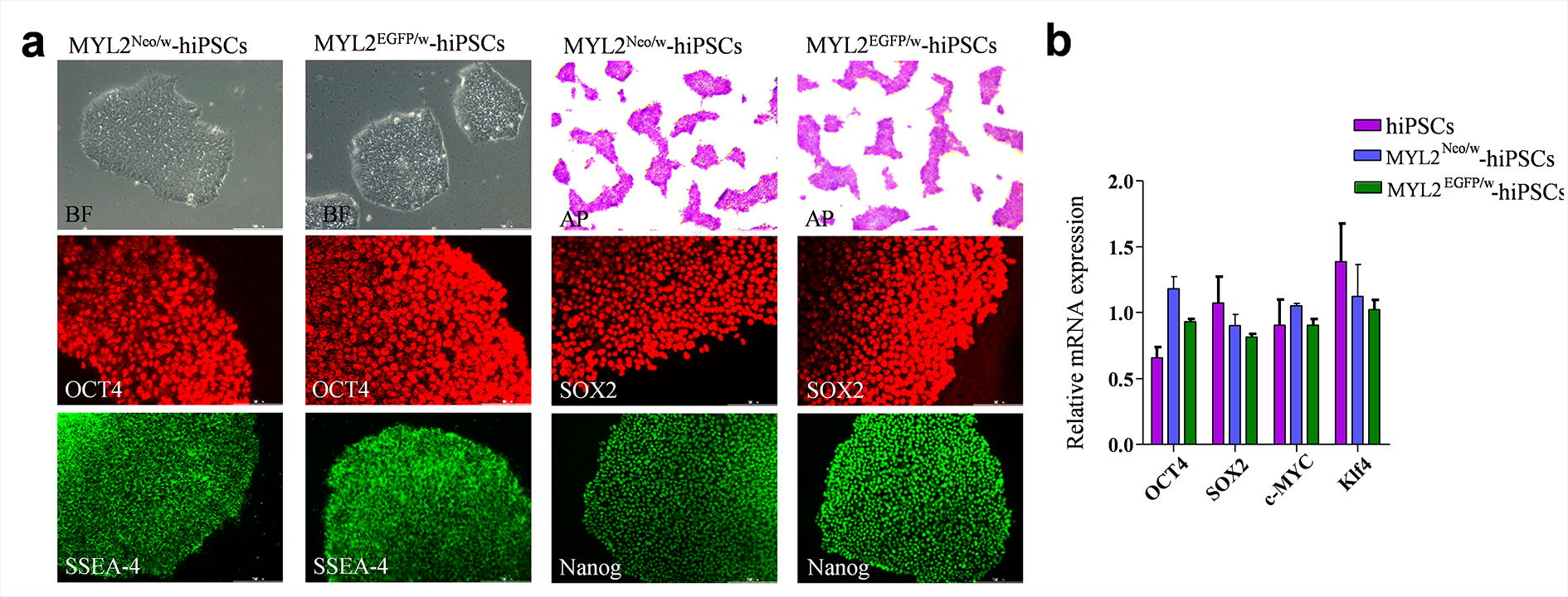

Supplement: Supplementary file 12 — Showing analyses of pluripotency in representative hiPSC clones. a, b MYL2Neo/w-hiPSCs and MYL2EGFP/w-hiPSCs exhibited similar morphologies, alkaline phosphatase activities, and expression levels for the pluripotent genes OCT4, SOX2, Klf4, and c-MYC to those in wildtype hiPSCs. Scale bars, 200 μm. (JPG 266 kb) [file 13287_2017_651_MOESM12_ESM.jpg]

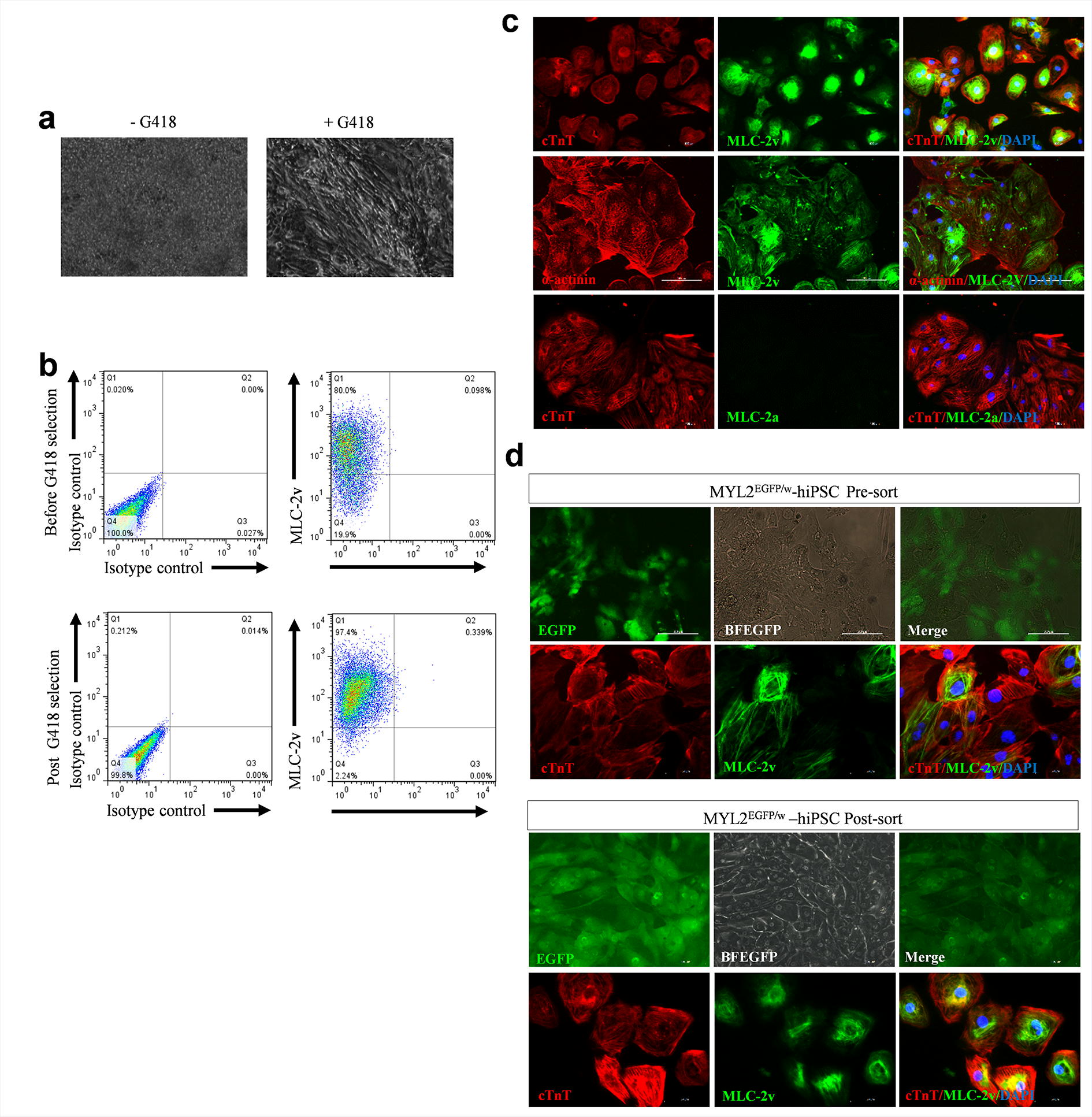

Supplement: Supplementary file 13 — Showing enrichment of human MLC-2v-positive early ventricular cardiomyocytes from hiPSCs. a, b Adding G418 after cardiac differentiation of MYL2Neo/w-hiPSCs successfully selected cardiomyocytes and markedly increased the percentage of MLC-2v-positive cells to ~ 98%. c Immunostaining also showed that, after G418 selection, MLC-2v-positive cardiomyocytes were enriched while MLC-2a-positive cardiomyocytes were almost completely removed. d FACS sorting based on GFP expression after cardiac differentiation of MYL2EGFP/w-hiPSCs successfully enriched pure MLC-2v-positive early ventricular cardiomyocytes. Almost 100% of these EGFP+ cells showed strong MLC-2v expression in cTnT-positive cardiomyocytes. (JPG 475 kb) [file 13287_2017_651_MOESM13_ESM.jpg]

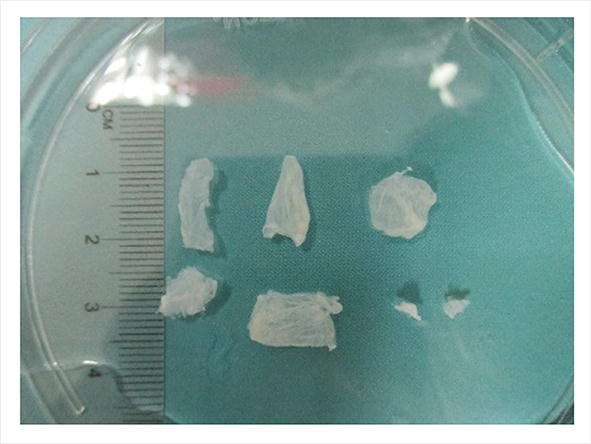

Supplement: Supplementary file 16 — Showing the different sizes and shapes of the engineered ventricular muscles. (JPG 31 kb) [file 13287_2017_651_MOESM16_ESM.jpg]
