# Supplementary material for: Engineering human ventricular heart muscles based on a highly efficient system for purification of human pluripotent stem cell-derived ventricular cardiomyocytes
Source: Stem Cell Res Ther. 2017 Sep 29;8:202. doi: 10.1186/s13287-017-0651-x (PMC5622416; doi:10.1186/s13287-017-0651-x)
Supplement: Supplementary file 4 — Presenting summary of off-target analysis for the MYL2 targeting locus. Ten potential TALEN off-target sites were estimated by the TALENoffer software. All 10 sites were amplified by PCR and sequenced. (DOCX 16 kb) [file 13287_2017_651_MOESM4_ESM.docx]

| **Rank** | **Chr** | **Position 1** | **Position 2** | **Gene** | **Score** | **Full-site** | **Off-target** |
| --- | --- | --- | --- | --- | --- | --- | --- |
| 1 | 20 | 9656435 | 9656477 | PAK7 | -1.487 | TGGATAACAAACAAATTAgtcaactgataccatgtgtcttgcTGCTTATTCTGTTAGTCA | Negative |
| 2 | 19 | 7678460 | 7678495 | CAMSAP3 | -1.509 | TCCCTCCCAAACAAGGTAtgggtggccggacaaggGGGATAGATAGATGTGGGGA | Negative |
| 3 | 9 | 76821743 | 76821773 | LOC101927329 | -1.521 | TGGCTAAAAGACCAAGTAacaacagagagaGTGAGAGAGAGAAGGAGGGA | Negative |
| 4 | 11 | 115422646 | 115422688 | - | -1.566 | TTGGTATCAGACAAGATAatctctagggtctcttctaatcttTACTTTGTCTGTGAGTTG | Negative |
| 5 | 4 | 61750192 | 61750225 | - | -1.570 | TTACTAGAAAACAAAGTCagcaaccttgcccctTACCTTTTCTGTTTGCCA | Negative |
| 6 | 3 | 159854026 | 159854070 | IL12A-AS1 | -1.573 | TGCCCCCATCTCGCTCCCCCaaggttttcttgaagcccctttcaTTGGGTTTGAGATGGGAGGA | Negative |
| 7 | 2 | 84474588 | 84474629 | - | -1.574 | TGACCTCATCACTCTTCACAgggaaagttgacattcttctcTACATGGTATGTTAGGCA | Negative |
| 8 | 18 | 42554317 | 42554355 | SETBP1 | -1.589 | AAGCTCAAAACCAAGGTAgctaagtaagtttggcaaggAGGGGAGGGAGATGAGAGTA | Negative |
| 9 | 5 | 149353096 | 149353136 | SLC26A2 | -1.601 | TCCCCTCATCTCTCTCTCCCgttccccccacatatatatgGTGCTGGTCTGCTGGCCT | Negative |
| 10 | 12 | 87168166 | 87168206 | PRKG1 | -1.601 | CTCTCTCCTCTCTCTCTCTCtctctctctctctttctttcTTTCTTTTCTGTTAGCCA | Negative |
