# Supplementary material for: Engineering human ventricular heart muscles based on a highly efficient system for purification of human pluripotent stem cell-derived ventricular cardiomyocytes
Source: Stem Cell Res Ther. 2017 Sep 29;8:202. doi: 10.1186/s13287-017-0651-x (PMC5622416; doi:10.1186/s13287-017-0651-x)
Supplement: Supplementary file 9 — Presenting whole-cell patch clamp recordings of action potentials of ventricular-like, atrial-like, and nodal-like cells produced from day 30 MYL2Neo/w-derived cardiomyocytes before and post G418 selection. (DOCX 17 kb) [file 13287_2017_651_MOESM9_ESM.docx]

|  | | MDP  (mV) | Overshoot  (mV) | APA (mV) | APD  (ms) | APD90 (ms) | APD70 (ms) | APD50 (ms) | Vmax-D (V/s) | Beating rate (Beat per minute) | APD90 /APD50 | SD of Interspike Interval | % Total |
| --- | --- | --- | --- | --- | --- | --- | --- | --- | --- | --- | --- | --- | --- |
| **V-like** | Bath  (n=13) | -61.6±1.6 | 34.8±2.2 | 96.5±3.1 | 331.1±53.2 | 264.1±52.0 | 248.9±51.2 | 232.6±49.6 | 6.8±0.7 | 90.4±12.2 | 1.17±0.02 | 129.1±64.3 | 56.5 |
|  | G418  (n=16) | -56.8±1.1 | 38.4±1.3 | 95.1±1.9 | 260.6±12.5 | 211.4±12.0 | 195.5±11.9 | 179.4±11.5 | 7.4±0.6 | 129.4±9.3 | 1.19±0.01 | 25.2±10.1 | 80.0 |
| **A-like** | Bath  (n= 5) | -63.5±1.6 | 26.6±0.7 | 90.1±1.4 | 171.0±27.9 | 110.4±17.7 | 94.6±14.9 | 81.7±12.5 | 5.0±0.9 | 140.8±24.9 | 1.35±0.02 | 31.0±9.8 | 21.7 |
|  | G418  (n=3) | -56.9±6.5 | 32.1±2.8 | 89.0±3.7 | 230.2±62.0 | 166.8±48.6 | 145.8±43.3 | 125.1±37.5 | 10.9±5.4 | 159.7±48.9 | 1.34±0.02 | 24.4±18.0 | 15.0 |
| **N-like** | Bath  (n=5) | -57.3±2.1 | 10.1±1.9 | 67.4±3.9 | 293.0±45.4 | 159.6±38.2 | 116.8±35.2 | 89.7±29.0 | 2.4±0.6 | 90.2±18.1 | 2.49±0.67 | 37.7±6.7 | 21.7 |
|  | G418  (n=1) | -49.5 | 16.8 | 66.3 | 136.3 | 80.6 | 66.5 | 55.7 | 2.1 | 183 | 1.45 | 6.6 | 5.0 |
