# Supplementary material for: Engineering human ventricular heart muscles based on a highly efficient system for purification of human pluripotent stem cell-derived ventricular cardiomyocytes
Source: Stem Cell Res Ther. 2017 Sep 29;8:202. doi: 10.1186/s13287-017-0651-x (PMC5622416; doi:10.1186/s13287-017-0651-x)
Supplement: Supplementary file 11 — Presenting whole-cell patch clamp recordings of action potentials of ventricular-like, atrial-like, and nodal-like cells produced from day 30 MYL2EGFP/w-derived cardiomyocytes before and post FACS sorting. (DOCX 16 kb) [file 13287_2017_651_MOESM11_ESM.docx]

|  | | MDP  (mV) | Overshoot (mV) | APA  (mV) | APD (ms) | APD90 (ms) | APD70 (ms) | APD50 (ms) | Vmax-D (V/s) | Beating rate (Beat per minute) | APD90 /APD50 | SD of Interspike Interval | % Total |
| --- | --- | --- | --- | --- | --- | --- | --- | --- | --- | --- | --- | --- | --- |
| **V-like** | Bath  (n=13) | -61.6±1.6 | 34.8±2.2 | 96.5±3.1 | 331.1±53.2 | 264.1±52.0 | 248.9±51.2 | 232.6±49.6 | 6.8±0.7 | 90.4±12.2 | 1.17±0.02 | 129.1±64.3 | 56.5 |
|  | eGFP  (n=21) | -63.8±0.7 | 42.3±1.2 | 106.1±1.6 | 468.8±35.9 | 295.0±21.6 | 274.6±21.2 | 254.0±20.6 | 17.7±1.5 | 68.0±12.9 | 1.18±0.02 | 202.3±58.5 | 91.3 |
| **A-like** | Bath  (n= 5) | -63.5±1.6 | 26.6±0.7 | 90.1±1.4 | 171.0±27.9 | 110.4±17.7 | 94.6±14.9 | 81.7±12.5 | 5.0±0.9 | 140.8±24.9 | 1.35±0.02 | 31.0±9.8 | 21.7 |
|  | eGFP  (n= 2) | -63.0±1.8 | 39.4±3.5 | 102.5±5.4 | 315.9±147.5 | 173.1±63.9 | 145.8±53.1 | 124.2±44.7 | 26.4±3.8 | 152.5±63.5 | 1.39±0.01 | 31.0±16.1 | 8.7 |
| **N-like** | Bath  (n=5) | -57.3±2.1 | 10.1±1.9 | 67.4±3.9 | 293.0±45.4 | 159.6±38.2 | 116.8±35.2 | 89.7±29.0 | 2.4±0.6 | 90.2±18.1 | 2.49±0.67 | 37.7±6.7 | 21.7 |
|  | eGFP  (n=0) |  | | | | | | | | | | | 0.0 |
